# Integrated Molecular Informatics and Sensory-Omics Study of Core Trace Components and Microbial Communities in Sauce-Aroma High-Temperature Daqu from Chishui River Basin

**DOI:** 10.3390/foods15030599

**Published:** 2026-02-06

**Authors:** Dandan Song, Lulu Song, Xian Zhong, Yashuai Wu, Yuchao Zhang, Liang Yang

**Affiliations:** 1 School of Brewing Engineering, Moutai Institute, Renhuai 564501, China; songdd0330@163.com (D.S.); songlulu@mtxy.edu.cn (L.S.); zhongxian2025@163.com (X.Z.); zyc10271027@163.com (Y.Z.); 2 School of Food Science and Engineering, South China University of Technology, Guangzhou 510640, China; wyss995418706@163.com

**Keywords:** sauce-aroma high-temperature Daqu, core trace components, core microorganisms, Baijiu, molecular informatics and sensory omics

## Abstract

Flavor-relevant trace volatiles and microbial communities were examined in six sauce-aroma high-temperature Daqu samples. Headspace solid-phase microextraction coupled with gas chromatography–mass spectrometry (HS-SPME-GC-MS) quantified 210 trace volatile compounds across 14 chemical classes. Orthogonal partial least squares discriminant analysis (OPLS-DA) with variable importance in projection (VIP) screening was integrated with sensory scoring, correlation analysis, and molecular docking to an olfactory receptor model. Volatile profiles showed clear stratification in total abundance. Pyrazines dominated the high-total group. Tetramethylpyrazine served as a major driver. Sensory evaluation indicated that aroma explained overall quality best. (E)-2-pentenal and dimethyl trisulfide showed significant positive associations with aroma and overall scores. In the olfactory receptor, the polar residue module that provides directional constraints for Daqu odor activation was formed by Ser75, Ser92, Ser152, Ser258, Thr74, Thr76, Thr98, Thr200, Gln99, and Glu94. The hydrogen-bond or charge network was further reinforced by Arg150, Arg262, Asn194, His180, His261, Asp182, and Gln181. The core discriminant set comprised acetic acid, hexanoic acid, (E)-2-pentenal, nonanal, decanal, dimethyl trisulfide, trans-3-methyl-2-n-propylthiophane, 2-hexanone oxime, ethyl linoleate, propylene glycol, 2-ethenyl-6-methylpyrazine, 4-methylquinazoline, 5-methyl-2-phenyl-2-hexenal, and 1,2,3,4-tetramethoxybenzene. Sequencing revealed higher bacterial diversity than fungal. *Bacillus* and *Kroppenstedtia* were dominant bacterial genera. *Aspergillus*, *Paecilomyces*, *Monascus*, and *Penicillium* were major fungal genera. Correlation patterns suggested that *Bacillus* and *Monascus* were positively linked to acetic acid and 1,2,3,4-tetramethoxybenzene. Together, these results connected chemical fingerprints, sensory performance, receptor-level plausibility, and microbial ecology. Concrete targets are provided for quality control of high-temperature Daqu.

## 1. Introduction

Baijiu is a traditional distilled spirit that is produced from cereal grains through solid-state fermentation and distillation. It is widely regarded as the national liquor of China. It is also described as one of the six major distilled spirits worldwide. Among the aroma types of Baijiu, sauce-aroma Baijiu is considered a highly representative type [1,2,3,4]. Its style is characterized by a prominent sauce-like aroma, elegant finesse, a full-bodied mouthfeel, a long finish, and a persistent aroma in the empty glass. Consumer demand remains strong. In recent years, the sauce-aroma sector has expanded rapidly and become one of the most prominent sectors in the industry. In 2024, national sauce-aroma Baijiu production capacity was approximately 650,000 kL. Sales revenue was approximately 240 billion CNY, and profit was approximately 97 billion CNY. In 2024, the total output of the Baijiu industry above designated size was 4,145,000 kL, with sales revenue of 796.38 billion CNY and total profit of 250.87 billion CNY. Based on these figures, sauce-aroma Baijiu contributed about 15.7% of capacity, about 30.1% of industry revenue, and about 38.7% of industry profit (https://assets.kpmg.com/content/dam/kpmg/cn/pdf/zh/2025/06/mid-term-research-report-on-the-chinese-baijiu-market-2025.pdf, accessed on 27 January 2026). The Chishui River Basin has attracted multiple leading sauce-aroma Baijiu producers [5,6,7]. Four sauce-aroma enterprises have annual revenues above 10 billion CNY. The distinctive quality of sauce-aroma Baijiu and rising market demand keep the sector at the center of attention in the Baijiu industry.

Sauce-aroma Baijiu brewing is exemplified by the production area in the Chishui River Basin of Guizhou Province, with Moutai Town as the core [8,9]. This region is often described as a prime zone for sauce-aroma Baijiu production [5]. The traditional process is distinctive and highly complex [10]. It requires a 1-year cycle with two feedings, nine steamings, eight fermentation rounds, and seven liquor collections. The process is known in the industry as “12987” [11,12,13]. Within this long process, sauce-aroma high-temperature Daqu is regarded as the core of the brewing [14]. Sauce-aroma high-temperature Daqu is produced from grains such as wheat [5]. It relies on natural inoculation under open conditions and is formed under a high-temperature environment [13]. The maximum temperature during Daqu production can exceed 60 °C. Sauce-aroma high-temperature Daqu serves as the main carrier of microorganisms required for fermentation. It also serves as a saccharifying and fermenting agent [14,15]. It is an important source of flavor precursors. Many studies have indicated that sauce-aroma high-temperature Daqu plays a key role in the formation of the characteristic flavor of sauce-aroma Baijiu [8]. Microbial consortia enriched in Daqu provide functional strains and enzyme systems for sauce-aroma fermentation. Daqu quality is directly linked to the yield and quality of the final Baijiu. Dominant microorganisms in Daqu are often the core functional microorganisms for aroma formation and enzyme production [16,17,18,19,20,21,22]. They directly influence the formation of the final flavor profile.

The Chishui River Basin, with Moutai Town as the core, is distinguished by a river-valley basin setting and a long-established brewing landscape that together create a relatively closed and stable local microclimate, typically characterized by warm and humid conditions with comparatively low wind speed, which favors the persistence, dispersal, and long-term domestication of fermentation-related microorganisms in and around production spaces [23,24]. In addition to climatic stability, regional hydrogeochemical features further strengthen this uniqueness, as the basin has been reported as the core area for sauce-aroma Baijiu, and its water chemistry (for example, near-neutral pH and abundant trace elements) is considered supportive of microbial growth and metabolic activity, thereby providing a consistent ecological backdrop for open fermentation [23,25]. Under such conditions, the open and highly interactive production ecology of sauce-aroma Baijiu becomes especially consequential: high-temperature Daqu making and subsequent solid-state fermentation are extensively exposed to ambient air and surrounding environments, allowing environmental microorganisms to repeatedly enter and be selected across cycles, and accumulating a place-linked microbial reservoir that is difficult to reproduce in non-local settings [26]. Recent evidence from source-tracking and environmental microbiome investigations supports that microbes from surrounding matrices can contribute to Daqu community assembly and that airborne microbiomes in the Chishui River Basin core production areas show structured [27,28,29,30,31,32] community patterns shaped by environmental factors, reinforcing the concept that regional environmental ecology is a key driver of microbial community stability and, ultimately, characteristic flavor expression [8]. Collectively, the coupling of the basin-specific geography and microclimate, water- and soil-related geochemical context [6], and centuries of open, multi-round brewing practices forms a distinctive microbial “terroir” in Moutai Town, which is widely regarded as a fundamental basis for sauce-aroma Baijiu flavor formation and a major reason why authentic sensory typicity is challenging to replicate outside the core Chishui River Basin region [33,34,35,36,37,38,39,40].

Under the coordinated action of diverse microorganisms in solid-state fermentation, Chinese Baijiu develops a highly complex chemical matrix, yet only a subset of compounds ultimately drives sensory identity. In Baijiu manufacture, Daqu is not a passive additive but a core starter that supplies functional microbiota, enzyme systems, and flavor precursors, enabling the characteristic “simultaneous saccharification and fermentation” strategy and supporting the initiation and stability of fermented-grain (Jiupei) fermentation [13]. For sauce-aroma Baijiu in particular, the workflow includes mixing fermented grains with Daqu and water, performing high-temperature stacking on the workshop floor for several days, and then transferring the stacked grains to a mud-sealed cellar for extended anaerobic fermentation before distillation, followed by grading and storage [25]. Because Daqu governs the early establishment of microbial consortia and enzyme activities that drive starch degradation [41], amino acid release, and precursor formation, variations in Daqu quality can propagate through fermentation and translate into differences in product quality and manufacturing robustness, which is why the use of high-quality Daqu is essential for producing commercially acceptable Baijiu [16,17,18,20,21,33,42].

High-temperature Daqu should also be distinguished clearly from other starter types. Daqu is commonly categorized by its maximum fermentation temperature into high-temperature Daqu (typically associated with sauce-aroma or Maotai-flavor Baijiu), medium-temperature Daqu (commonly used for strong-aroma Baijiu), and low-temperature Daqu (commonly used for light-aroma Baijiu), reflecting different incubation regimes that select different microbial communities and functional profiles [13,27]. Sauce-aroma high-temperature Daqu is characteristically produced from wheat and incubated at higher temperatures, often reaching 60–65 °C under open conditions, which enriches thermotolerant microorganisms and promotes the formation of enzymes and metabolites that support the subsequent high-temperature stacking and cellar fermentation stages typical of sauce-aroma Baijiu production [25]. Given the long production cycle and the strong dependence of sauce-aroma-style expression on microbial metabolism, a systematic framework that links Daqu microbiota with characteristic trace aroma compounds is needed. Therefore, this study focuses on identifying key functional microorganisms and core flavor-related trace components in sauce-aroma high-temperature Daqu and integrating chemical, microbiological, and sensory evidence to improve interpretability and practical relevance [13,27].

Based on this context, an integrated molecular informatics and sensory-omics strategy was applied to sauce-aroma high-temperature Daqu from the Chishui River Basin. The objective was to systematically identify key odor-active substances and core functional microorganisms. The material basis and microbial mechanisms underlying the characteristic aroma were also examined. Molecular-level flavor analysis was coupled with human sensory evaluation. The internal links among the regional environment, process parameters, and flavor characteristics were thereby clarified more comprehensively. Molecular informatics enabled the acquisition of large-scale volatile data. Sensory-omics evaluation supported assessment of contributions to overall aroma. This strategy was expected to pinpoint the most representative odorants in Daqu and to locate the core functional microorganisms that drove their formation. The outcomes were expected to support the optimization and standardization of traditional sauce-aroma processes. Brewing practice was expected to shift from experience-based operation toward more precise and rational guidance.

## 2. Materials and Methods

### 2.1. Samples and Reagents

**Figure 1:**
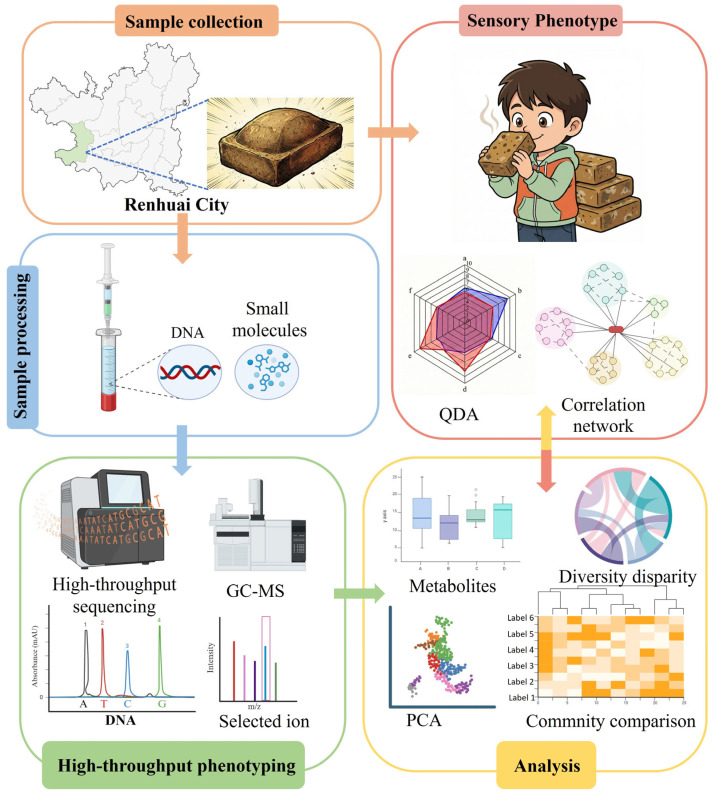
Workflow of this study. QDA: quantitative descriptive analysis.

Samples were collected in Moutai Town, Renhuai, Zunyi, Guizhou Province, located in the core production area of sauce-aroma Baijiu in the Chishui River Basin. Six distilleries were randomly selected along the river continuum (from upstream to downstream) and were coded as Daqu_A, Daqu_B, Daqu_C, Daqu_D, Daqu_E, and Daqu_F. Within each distillery, 3 Daqu rooms were randomly selected, and within each Daqu room, 3 Daqu bricks were randomly selected (6 distilleries × 3 rooms × 3 bricks, n = 54 bricks in total). Bricks from the same distillery were crushed and homogenized, and 3 representative composite samples were obtained per distillery, resulting in 18 representative samples overall (n = 18). To facilitate parallel multi-omics measurements and clearly distinguish experimental variants and analytical branches, a study flowchart summarizing sampling, pooling, and downstream processing was added (Figure 1).

High-temperature sauce-aroma Daqu was produced under routine commercial solid-state fermentation conditions using 100% wheat as the sole substrate. During the stacking stage, the internal fermentation temperature increased to a peak of 60–65 °C, which was consistent with the typical peak-temperature window reported for high-temperature Daqu manufacture. Throughout stacking, the relative humidity was maintained at 10–40% (i.e., under low-humidity/low-moisture conditions), aligning with the general low-moisture nature of Daqu solid-state fermentation, where moisture content is reported to decline substantially during production. The solid-state fermentation lasted for exactly 40 days, matching the commonly described ~40-day high-temperature fermentation period for Jiang-/sauce-flavor Daqu prior to subsequent storage/maturation. Finally, all samples were collected at the mature harvest stage (post-maturation) to ensure that the profiled microbial communities and volatile signatures represent the final, stabilized starter state actually used for Baijiu brewing after fermentation and storage.

Each representative sample was divided into 2 portions: one portion was stored at 4 °C for metabolome evaluation, and the other portion was stored at −80 °C for microbial community analysis. All authentic standards had a minimum purity of 97% and were purchased from J & K (Beijing, China) and Sigma-Aldrich (Shanghai, China). 4-octanol, 2-ethylbutyric acid, and amyl acetate were used as internal standards, and ultrapure water was obtained from a Milli-Q system.

### 2.2. Experimental Procedures

#### 2.2.1. Pretreatment of Sauce-Aroma High-Temperature Daqu

Volatile compounds were enriched and extracted by HS-SPME. A 2 g Daqu sample was weighed into a 20 mL headspace vial containing 6 mL saturated NaCl solution. High-temperature Daqu samples were collected as mature (post-maturation) Daqu bricks from routine commercial production, then crushed and homogenized to obtain representative composite materials. The samples were handled “as received” on a wet-weight basis (i.e., no additional oven drying or dehydration treatment was applied after collection), consistent with how mature Daqu is used directly as a starter in Baijiu brewing. A 10 μL mixed standard solution was added. The final concentration was 20 μg/mL. The vial was capped and sealed. Equilibration was performed at 40 °C for 20 min. A 50/30 μm DVB/CAR/PDMS fiber was used. The fiber was exposed to the vial headspace. Extraction was performed with stirring at a temperature below 40 °C for 40 min. Desorption was performed immediately in the GC inlet at 250 °C for 5 min.

#### 2.2.2. HS-SPME and GC-MS Conditions for Sauce-Aroma High-Temperature Daqu

Volatile compounds were analyzed by a GC-MS system (7890B GC System, 5977A MSD). A DB-FFAP capillary column was used (60 m × 250 μm × 0.25 μm, Agilent Technologies, Santa Clara, CA, USA). Helium was used as the carrier gas with a purity of 99.999%. The flow rate was kept constant at 2.0 mL/min. The inlet temperature was set to 250 °C. The oven program was as follows. The initial temperature was held at 40 °C for 3 min. The temperature was increased to 150 °C at 2 °C/min and held for 2 min. The temperature was increased to 230 °C at 7 °C/min and held for 8 min. The transfer line temperature was set to 250 °C. The ion source temperature was set to 230 °C. EI was operated at 70 eV. The scan range was *m*/*z* 35–450 amu. Injection was performed in splitless mode. Qualitative identification was performed by matching mass spectra to the NIST (version, 2020) library. Quantification was performed by the internal standard method.

#### 2.2.3. Molecular Docking

Molecular docking was performed in Maestro 14.5. The receptor was the cryo-EM structure of the human olfactory receptor OR51E2 deposited in the RCSB PDB as 8F76. The structure was an active conformation with a propionate-bound fatty acid odorant. It was also in complex with miniGs. Receptor preparation was performed with the Protein Preparation Wizard workflow. Hydrogen atoms were added. Bond orders and formal charges were assigned. Missing side chains were completed. The hydrogen-bond network was optimized. Restrained minimization was performed under the OPLS4 force field to reduce local strain while maintaining the global conformation of the transmembrane helices. Crystallization additives and free water molecules unrelated to the binding cavity were removed. Structural water molecules that were located deep in the pocket and contributed to bridging polar interactions were retained when cavity geometry was not affected [43,44,45,46,47].

Ligand conformations and protonation states were generated by LigPrep with Epik. Three-dimensional structures were standardized. Major ionization states and tautomers within the specified pH window were enumerated. Relevant stereoisomers were retained to cover possible orientations and conformational heterogeneity. The receptor grid was defined by using the co-crystallized ligand position in 8F76 as a geometric reference. The center and boundary of the orthosteric pocket were defined within the transmembrane bundle. The grid box covered the main cavity and the adjacent narrow channel. Hydrophobic coordination and polar anchoring were both sampled. Docking was performed with the standard Glide small-molecule protocol [46,48]. The ligand vdW scaling factor was set to 0.80. A partial charge cutoff of 0.15 was applied to improve tolerance for flexible ligands and to increase pose diversity. Sampling was performed in SP mode with post-docking minimization and pose rescoring. Top-ranked poses were rechecked in XP mode when needed. Docking score and Emodel were considered during evaluation. GlideScore approximated binding free-energy trends. Emodel supported the selection of reasonable poses among multiple poses of the same ligand. The reported ecoul and evdw terms were used to separate electrostatic and van der Waals contributions. The final poses were analyzed in Maestro. Hydrogen bonds, salt bridges, π-related interactions, and hydrophobic contacts were recorded. These results supported residue-level mechanistic interpretation in subsequent analyses [47,49].

#### 2.2.4. Sensory Evaluation of Sauce-Aroma High-Temperature Daqu

The sensory attributes of mature sauce-aroma high-temperature Daqu were considered an intuitive signal of whether Daqu making met the process requirements. They were also regarded as an early indicator of subsequent style stability in sauce-aroma Baijiu. Because aroma and appearance were easily affected by ambient odors, noise, and lighting, sensory evaluation was conducted under controlled conditions. The facility followed general principles for a sensory analysis laboratory. It included separate functional areas, such as a testing area and a sample preparation area. This layout reduced the impact of sample preparation on air quality and background odors in the testing area. Environmental controllability and consistency were emphasized during evaluation. Temperature and humidity in the testing area were set to ensure assessor comfort and to meet sample handling needs. Noise was controlled at 40 dB or lower. Lighting was adjustable and was typically set at 6500 K. Illuminance was maintained within 200 lx to 400 lx. External odors were controlled by ventilation and filtration. Residual odors were minimized to reduce carryover between samples.

A trained sensory panel was formed. The panel consisted of 10 assessors, including 5 males and 5 females, aged 20–29 years. Routine training focused on descriptor recognition, scale use, and sensory memory retention. These measures ensured comparability in discrimination for samples evaluated within the same batch. Samples were presented with random codes and a balanced serving order. Within the same session, assessors completed visual inspection, aroma evaluation, and cross-section observation. During evaluation, surface color and mold growth distribution were examined first. Representative bricks were then fractured to assess cross-section color uniformity, pore structure, and mycelial appearance. Aroma was assessed with brief and repeated sniffs to capture dominant notes and potential off-odors. A weighted scoring method was applied. Surface color, aroma, and cross-section were used as core dimensions. A higher weight was assigned to aroma to align with practical needs in quality assessment of sauce-aroma high-temperature Daqu (Table 1).

### 2.3. Metagenomic Sequencing

Total DNA in Daqu was extracted using the QIAGEN DNeasy mericon Food Kit (QIAGEN, Hilden, Germany) according to the manufacturer’s instructions. DNA quality was evaluated by 1% agarose gel electrophoresis. DNA concentration was measured with a NanoDrop™ One spectrophotometer (Thermo Fisher Scientific, Waltham, MA, USA). This ensured suitability for subsequent sequencing. The extracted DNA was used to construct metagenomic sequencing libraries. All libraries were sequenced on an Illumina NovaSeq X Plus platform (Illumina Inc., San Diego, CA, USA). Sequencing was completed by Shanghai Majorbio Bio-pharm Technology Co., Ltd. (Shanghai, China). A 150 bp paired-end mode was applied to provide sufficient coverage for downstream microbial community structure analysis.

Quality-filtered reads were assembled using MEGAHIT (v1.1.2). Short reads were merged into longer contigs by a fast assembly algorithm. Contigs were filtered by length. Contigs with a length of at least 300 bp were retained as the final assembly. Open reading frames (ORFs) were predicted on the assembled contigs using Prodigal (v2.6.3). Prodigal identified gene regions across different taxa. Putative protein-coding sequences were extracted. Only ORFs with a length of at least 100 bp were retained to improve prediction reliability.

A non-redundant gene catalog was constructed using CD-HIT (v4.7) to reduce sequence redundancy and improve analytical efficiency. Gene sequences were clustered at 90% sequence identity and 90% coverage. Each cluster contained a representative sequence. This provided a streamlined and reliable gene catalog for subsequent analyses.

### 2.4. Statistical Analysis

Statistical computing was performed mainly in R (v4.5.x, R Foundation). Mixed-effects models were fitted using lme4. Estimated marginal means and post hoc contrasts were obtained using emmeans. Reproducible hypothesis testing and regression analyses were cross-checked in Python (v3.15.0). One-way ANOVA and correlation analyses were performed using IBM SPSS Statistics 24. Adjusted *p*-values were reported. Data visualization and curve fitting were performed in GraphPad Prism 9 and OriginPro 2021. Heatmaps and clustering plots were generated using TBtools(v2.119).

## 3. Results and Discussion

### 3.1. Volatile Components in Daqu Determined by HS-SPME-GC-MS

As shown in Table S1, HS-SPME-GC-MS was used to qualitatively and quantitatively determine 210 volatile trace components in six Daqu groups (A–F; n = 3 per group). Fourteen chemical classes were covered. Aldehydes and ketones, esters, alcohols, aromatic compounds, and pyrazine were among the most abundant classes in high-temperature sauce-aroma Daqu by number of compounds.

At the total level, the summed concentration of volatile components in the six Daqu groups showed clear stratification. All concentrations were reported in µg/g. The mean value of group A was 303.69 ± 12.51. Group B was 199.41 ± 44.73. Group C was 123.76 ± 6.63. Group D was 479.11 ± 37.76. Group E was 498.30 ± 43.97. Group F was 467.03 ± 43.77. One-way ANOVA indicated a significant difference in total volatile concentration among groups (*p* < 0.05). Multiple comparisons showed no significant differences among D–F (*p* > 0.05). These three groups were significantly higher than A, B, and C (*p* < 0.05). Group A was significantly higher than B and C (*p* < 0.05). No significant difference was observed between B and C (*p* > 0.05). This result indicated a high-total group (D, E, and F), an intermediate group (A), and a low-total group (B and C) for the overall accumulation of volatile products in the sauce-aroma high-temperature Daqu.

After total abundance was decomposed by class, differences in dominant classes became more concentrated. Groups D, E, and F were dominated by pyrazines. The proportions of pyrazines in total volatiles reached 65.69%, 57.54%, and 54.03%, respectively. Group A also showed a high proportion of pyrazines at 46.44%, but the overall intensity was lower than that of D, E, and F. In contrast, the main contributions in group C were from other compounds and alcohols. Their proportions were 28.43% and 27.44%, respectively. Pyrazines accounted for only 10.54%. Statistical testing at the class level showed significant differences among samples for pyrazines, alcohols, fatty acids, aldehydes and ketones, and esters (*p* < 0.05). Only the total phenols did not differ significantly (*p* = 0.136). In the stacked solid-state fermentation system of sauce-aroma high-temperature Daqu, volatile phenols often showed a non-significant pattern. This pattern did not imply a weak flavor role. It reflected buffered net accumulation driven by formation and consumption mechanisms. Key precursors of phenols were often cell wall-bound phenolic acids such as ferulic acid. These precursors required release by microbial esterase systems. Volatile phenols such as 2-methoxy-4-vinylphenol could then be formed via phenolic acid decarboxylases. This route had enzymatic support in bacteria and yeasts. Bacillus was also reported to decarboxylate ferulic acid to 4-vinylguaiacol. However, the high-temperature and oxygen-rich solid-state environment of Daqu also enhanced the subsequent conversion and fate of volatile phenols. Oxidative enzymes could catalyze one-electron oxidation and coupling polymerization. This shifted monomeric phenols toward higher polymers. As a result, free phenolic monomers captured by headspace sampling did not accumulate readily. In addition, 4-vinylguaiacol and its precursors could exert inhibitory effects on microorganisms. Prior studies also suggested that conversion yield and final concentration could be constrained by toxicity and metabolic stress. This further reduced the magnitude of between-sample differences. Even when microecology and metabolic intensity differed among Daqu samples, phenol formation was constrained by precursor supply. Phenol depletion was also reinforced by oxidative polymerization and metabolic conversion. Group differences, therefore, remained difficult to distinguish [50,51,52,53,54,55,56,57]. A *p*-value above 0.05 was consistent with the dynamic behavior of phenols in sauce-aroma high-temperature Daqu.

At the individual-compound level, differences in pyrazines were mainly driven by tetramethylpyrazine. The mean levels of tetramethylpyrazine in A, B, C, D, E, and F were 112.13, 12.48, 4.77, 258.24, 220.80, and 195.80, respectively. This compound accounted for a high proportion in both total volatiles and within pyrazines. Its proportions within pyrazines reached 79.51% in A, 82.05% in D, 77.01% in E, and 77.59% in F. Its proportions in total volatiles were 36.92%, 53.90%, 44.31%, and 41.92%, respectively. The difference among Daqu groups was significant (*p* < 0.05). The trend was consistent with the class-level difference for pyrazines. In addition, 2,3,5-trimethylpyrazine remained at high levels in D, E, and F at 31.98, 36.82, and 36.09. These values were significantly higher than B and C at 5.32 and 4.06. The difference was also significant (*p* < 0.05). These compounds jointly formed a pyrazine framework for the high-total group.

For alcohols, the ranking across the six groups differed from that of pyrazines. The total alcohols were highest in F at 69.09. This level was significantly higher than most comparisons with A, B, C, D, and E (*p* < 0.05). A representative compound was phenethyl alcohol. Its mean value in F reached 51.07. This level was significantly higher than A, B, C, and D (*p* < 0.05). Group D was also significantly lower than E (*p* < 0.05). Phenethyl alcohol was often associated with floral or honey-like notes in prior studies on alcoholic beverages and Daqu making. It therefore provided strong interpretive value for flavor differences among Daqu samples.

Fatty acids highlighted the distinctive feature of group A. The total fatty acids in A were 30.37. This value was significantly higher than B, C, D, and F. It was also significantly higher than E (*p* < 0.05). Hexanoic acid reached 18.43 in A and 13.20 in E. It was not detected in B, C, D, and F. This led to a very significant difference for this compound (*p* < 0.01). The branched-chain acid 3-methylbutanoic acid was detected in all groups. Its level differed significantly among groups (*p* < 0.05). Groups A, D, and E were relatively higher, and F was lower. Because fatty acids and their esterification products often shape body and aftertaste, these acid-end differences provided a quantitative anchor for differences in flavor profiles.

The overall contribution of esters also showed a statistical difference (*p* < 0.05). Total esters were higher in A and F at 14.88 and 14.45. They were lower in C and D at 6.65 and 6.41. Ethyl hexadecanoate was among the major quantified ester peaks. It was close to 6.2 in A and F. It was about 2.07 in C. It was about 4.17 in D. The compound itself did not show a significant difference (*p* = 0.225). This suggested that ester differences were more likely driven by the combined effects of multiple medium- and low-abundance esters rather than by a single ester component.

Phenols were mainly contributed by 2-methoxy-4-vinylphenol. Its mean value was about 33.5 in B and E. It was about 27.75 in D. It was about 7.2 in A and F. It was only 0.18 in C. The difference did not reach significance (*p* = 0.133). This was consistent with the *p*-value for total phenols. The result was mainly influenced by increased within-group variation in B. Prior work indicated that volatile phenols often had high odor potency. They were also important in Daqu volatile profiles. Compounds such as 2-methoxy-4-vinylphenol were commonly discussed as key phenols. Stronger statistical discrimination for phenols would therefore require reduced within-group variance or increased replicate numbers. This would reduce cases in which a clear trend was observed but significance was not reached.

Sulfides showed a highly concentrated distribution. The mean sulfides in D reached 3.85. Groups A, C, and E were only 0.01 to 0.04. Sulfides were not detected in B and F. The class-level difference was highly significant (*p* < 0.05). This difference was almost entirely contributed by benzyl methyl sulfide. Its value was 3.85 in D. It was not detected in the other samples. Given the threshold sensitivity of sulfur volatiles, the enrichment in D should be regarded as a strong distinguishing signal. In the next step, specific trace components in each group are further analyzed to assess their importance.

### 3.2. Importance Analysis of Trace Components in Sauce-Aroma High-Temperature Daqu

As shown in File S1a, the model achieved an R2X of 0.934, an R2Y of 0.995, and a Q2 of 0.981. Values of R2 and Q2 above 0.5 indicated acceptable model performance. After 200 permutation tests, File S1 showed that the intercept of the Q2 regression line with the y-axis was below 0. This result indicated the absence of overfitting. Model validation was considered effective. The model was therefore considered suitable for analyzing key odor-active substances in sauce-aroma high-temperature Daqu. Trace components with VIP > 1 in the sauce-aroma high-temperature Daqu are shown in Table 2.

The Daqu-making temperature of sauce-aroma high-temperature Daqu could reach 60–65 °C. A multi-stage temperature rise and fall occurred under open stacking conditions. Microbial succession, enzyme release, and thermal reactions occurred in parallel. Systematic differences in volatile profiles among samples were therefore expected. Among trace components with VIP > 1, the highest contributors were not confined to a single class. They were jointly represented by nitrogen-containing heterocycles, fatty acids and related esters, lipid oxidation-related aldehydes and ketones, sulfur-containing compounds, and limited phenols and aromatic ethers. This pattern suggested that group discrimination mainly reflected differences in Maillard reaction intensity and pathways during high-temperature stacking. It also reflected differences in microbial supply of acids and esters. Additional differences were associated with lipid oxidation and amino acid degradation that affected aldehydes, ketones, and sulfur compounds.

In Table 2, the most characteristic markers of sauce-aroma high-temperature Daqu were pyrazines and derivatives. These included methylpyrazine, 2,3-dimethylpyrazine, 2,5-dimethylpyrazine, trimethylpyrazine, and tetramethylpyrazine. They also included highly substituted pyrazines with ethenyl, propyl, or acetyl substituents. Pyrazines typically convey roasted, nutty, cocoa-like, and toasted-cereal notes. This profile matched the stylistic basis of sauce-aroma Daqu, in which Daqu aroma and roasted notes were both emphasized. Tetramethylpyrazine was repeatedly highlighted in recent studies on sauce-aroma high-temperature Daqu and sauce-aroma Baijiu. It was regarded as a representative key flavor substance. Nut-like and baked notes could be imparted. Its formation was supported by both Maillard reactions and microbial synthesis during Daqu making, stacking fermentation, and solid-state fermentation. Table 2 also included 2-ethenyl-6-methylpyrazine, 2,3,5-trimethyl-6-propylpyrazine, and 2-acetyl-3,4,6-trimethylpyrazine. These higher substitution patterns suggested that differences were not limited to total pyrazines. Differences in substituent spectra were also likely. Substituent changes could markedly affect odor threshold, volatility, and aroma texture [14,25,58]. Highly substituted pyrazines often enhanced the depth of toasted and roasted nut-like notes. Certain samples could therefore present a stronger roasted base and a longer-lingering finish. Consistent with this view, studies on sauce-aroma Baijiu also reported that pyrazines were a key flavor framework. The pyrazine spectrum could diverge under different production areas or process conditions.

Typical aldehydes, ketones, and unsaturated aldehydes were also observed. This included acetaldehyde, hexanal, octanal, nonanal, decanal, trans-2-pentenal, trans-2-nonenal, 4-octanone, 2-nonanone, and 2-dodecanone. These compounds often produced a sharper aroma outline. Freshness and brightness could be provided at low levels. Oxidative notes and irritation could emerge at high levels. Trans-2-nonenal was often used as an indicator of lipid oxidation in food and beverage systems. It could present cucumber-like notes and cardboard-like oxidized off-odors. Its appearance as a VIP variable, therefore, suggested meaningful differences in lipid oxidation degree, precursor supply, or clearance rates across samples. Straight-chain aldehydes such as hexanal and octanal were more often associated with grassy and fatty notes. They could arise from autoxidation and enzymatic oxidation of raw material lipids. They could also be linked to the oxidative environment during high-temperature stacking. Strong aeration and heat release occurred during stacking. When oxygen participation increased, lipid oxidation chain reactions were more likely to proceed. Aldehydes could therefore become key variables for sample discrimination. In parallel, carbonyl compounds such as 2,3-butanedione and 2-hydroxy-3-pentanone also showed VIP > 1. They were often associated with buttery, creamy, and sweet notes in fermented foods. Softer roundness could be provided on top of a strong roasted base. Daqu aroma could therefore appear fuller and less harsh [22].

Acids and esters occupy a prominent position in Table 2. Acetic acid, propionic acid, hexanoic acid, octanoic acid, nonanoic acid, and multiple branched-chain acids were included. Paired esters included ethyl acetate, ethyl butyrate, ethyl valerate, ethyl octanoate, ethyl 2-methylbutanoate, ethyl 2-methylpropanoate, and ethyl 4-methylpentanoate. In sauce-aroma high-temperature Daqu, these variables often carried integrated information on microbial metabolism and enzymatic conversion. The acid side was commonly linked to carbon flux partitioning by lactic acid bacteria and other bacteria. It was also linked to amino acid degradation and lipid hydrolysis. Sensory cues could include acidic notes, fermentation impressions, sweaty notes, or cheese-like notes. Esters could partly soften acid sharpness. Fruity and sweet lift could be provided. Medium- and short-chain ethyl esters such as ethyl butyrate, ethyl valerate, and ethyl octanoate often contributed pineapple-like, apple-like, and composite fruity notes to Daqu and Baijiu systems. In sauce-aroma systems, fruity notes did not need to be exaggerated. Moderate fruity notes more often acted as support and elevation. Roasted Daqu aroma and sauce aroma could appear cleaner, with clearer layering. Acid–ester spectra as discriminant variables often implied differences in the balance between acid supply and esterification capacity. These differences could arise from microecological composition [34]. They could also reflect differences in enzyme activity windows shaped by stacking temperature curves.

Table 2 also shows phenols and aromatic ethers with clear smoky and spicy cues. 2-methoxy-4-vinylphenol is of particular interest. In many fermented and baked foods, this compound often presents clove-like and spicy notes with some smokiness. The roasted base can be extended toward a more penetrating spicy direction. In sauce-aroma high-temperature Daqu, such phenolic volatiles were often linked to the release of precursors such as ferulic acid and subsequent decarboxylation. They were also linked to thermal reaction pathways under high-temperature conditions. Co-occurrence with polymethoxy aromatic compounds such as 1,4-dimethoxybenzene, 1,2,3-trimethoxybenzene, and 1,2,3,4,5-pentamethoxybenzene suggested sweet, medicinal, or woody base notes. Thresholds and contribution patterns differed across compounds. Their appearance as VIP variables indicated measurable differences in woody sweetness and spiciness across samples. Sensory evaluation of sauce-aroma high-temperature Daqu often emphasized the purity of Daqu aroma and control of off-notes. Differences in this variable set, therefore, carried practical interpretive value. A cleaner spicy character could be indicated. Excessive smokiness or medicinal intensity could also occur under imbalance. Further judgment required integration with sensory descriptors and threshold calculations [59].

Sulfur-containing compounds were few but highly distinctive. Dimethyl trisulfide and benzyl methyl sulfide were particularly notable. Dimethyl trisulfide was often regarded as a typical high-impact sulfur odorant. Garlic-like, onion-like, and cooked cabbage-like notes could be expressed. Effects on overall aroma were often driven by a low threshold and strong impact. Stacking fermentation of sauce-aroma high-temperature Daqu occurred under high temperatures and complex nitrogen sources. Degradation of sulfur-containing amino acids and derivative reactions were therefore more likely to occur. High temperatures also promoted the formation of sulfur-containing heterocycles related to Maillard reactions. Studies on the identification of sauce-aroma high-temperature Daqu and related Baijiu reported that high-temperature fermentation produced more Maillard-derived products. These included pyrazines, aldehydes, furans, and sulfur-containing heterocycles such as thiophenes. This direction was consistent with the presence of thiophane signals in the VIP list. In sauce-aroma Daqu flavor construction, sulfur compounds often acted as finishing touches. Very low levels could enhance maturity and complexity. Levels above an appropriate range could shift toward irritation and unclean impressions. Sulfur compounds as discriminant variables, therefore, implied stable differences in sulfur precursor release, redox microenvironments, or downstream clearance mechanisms across samples.

Compounds with VIP > 1 also included furan and lactone structures related to lipid oxidation or thermal reactions. 2-pentylfuran and 5-butyl-4-methyl-2(3H)-furanone were representative. 2-pentylfuran was used in many foods as an indicator of lipid oxidation. Aroma could include green bean-like and green notes with nutty nuances. In Daqu aroma, such cues often appeared as a fresh, fatty character or a slight green edge. 5-butyl-4-methyl-2(3H)-furanone tended toward sweet and coconut-like lactone notes. Coconut-like cues were often noted in barrel-aged systems. Its presence as a VIP variable in Daqu could indicate differences in tendencies for lipid cleavage and cyclization toward lactones. A softer or more woody–sweet underpinning could therefore emerge at the base of Daqu aroma. These components were usually not the sole dominant aroma sources in sauce-aroma high-temperature Daqu. When combined with pyrazines, phenols, and acid–ester components, they often shaped whether Daqu aroma leaned toward a clean, roasted, nutty style or a sweeter, mature style. This shift could further affect the construction of the sauce-aroma framework during subsequent stacking fermentation [25,60].

Based on the clear separation in OPLS-DA and the structural composition of VIP compounds, differences among sauce-aroma high-temperature Daqu samples could be interpreted as coordinated shifts across multiple pathways. Differences in pyrazines and highly substituted derivatives indicated differences in Maillard reactions and nitrogen-related metabolism during high-temperature stacking. This direction aligned closely with the typical roasted Daqu aroma. Differences in aldehydes, ketones, and furans more strongly reflected differences in lipid oxidation and thermal cracking. Unsaturated aldehydes as oxidation markers suggested that some samples were more prone to oxidative edge notes. Sensory evaluation was still needed to judge whether this reflected freshness or oxidized off-odors. Differences in acids and esters reflected differences in the balance between acid formation and esterification capacity. These differences often determined fermentation thickness and the degree of fruity lift. Phenolic spiciness and smoky cues represented by 2-methoxy-4-vinylphenol, together with sulfur impact represented by dimethyl trisulfide, acted as variables that shaped flavor individuality. A stronger aroma texture could be created on top of a similar roasted framework.

It should be noted that VIP > 1 reflected contribution to supervised classification. It did not mean that each variable had significant differences in univariate tests. These VIP trace components will be correlated with sensory evaluation in subsequent analyses. A closed loop from mechanism to sensory performance and quality control will be constructed. Key odor-active substances in sauce-aroma high-temperature Daqu will be further screened.

### 3.3. Sensory Evaluation Analysis of Sauce-Aroma High-Temperature Daqu

**Figure 2:**
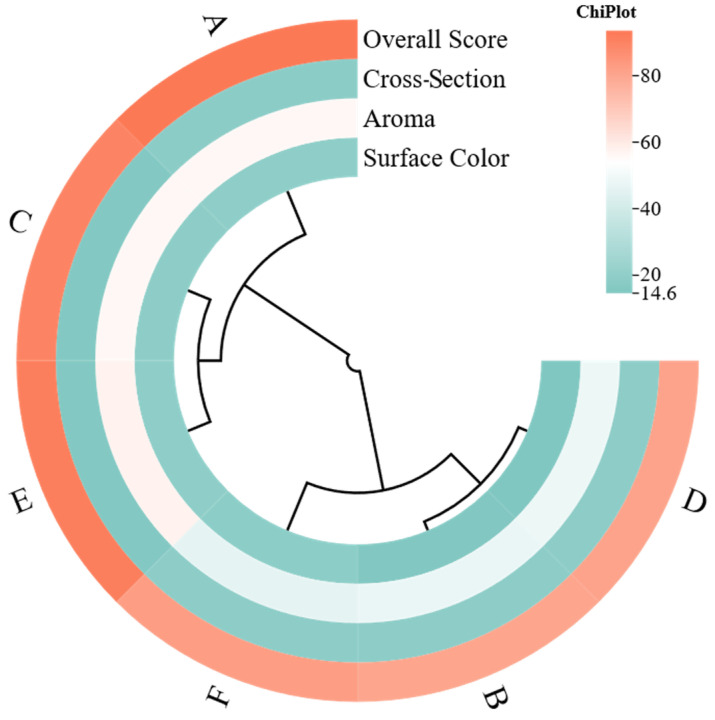
Overall sensory evaluation scores of sauce-aroma high-temperature Daqu.

Quantitative sensory evaluation was conducted for six groups of sauce-aroma high-temperature Daqu, covering surface color, aroma, cross-section, and overall score. As shown in Figure 2 and Table S2, the overall score ranged from 80.6 to 93.5 (A: 93.5; B: 80.6), with a between-group span of 12.9, indicating recognizable grade differences in overall sensory quality among Daqu from different sources or process conditions. The combinations across the four dimensions were not randomly distributed, and a relatively stable grouping pattern was observed, suggesting systematic sensory differences rather than incidental fluctuation. Surface color, aroma, and cross-section have also been widely used as core sensory indices in Daqu quality identification, and sensory evaluation is commonly regarded as a dominant basis for Daqu quality determination.

At the individual-dimension level, aroma showed the strongest discriminability, with scores ranging from 45.9 to 57.9 (range: 12.0), and its ranking closely matched the overall score. Groups A, C, and E showed high aroma scores (56.5, 56.3, and 57.9), with corresponding overall scores of 93.5, 90.2, and 91.8, whereas groups B, D, and F showed lower aroma scores (47.5, 48.2, and 45.9), with overall scores between 80.6 and 82.8. In Figure 2, the aroma band shows clear stratification in both the color scale and the dendrogram, consistent with the notion that aroma is the most critical quality cue in Daqu sensory systems and is often assigned a higher weight, thereby driving the overall score more strongly.

Surface color also differed across samples, ranging from 14.6 to 18.9 (span: 4.3). Groups A, C, E, and F clustered between 18.5 and 18.9, indicating closer alignment with typical high-quality Daqu in apparent maturity and integrated surface coloration, while groups B and D scored 14.8 and 14.6, suggesting relatively insufficient maturity and surface coloration. The surface color band in Figure 2 similarly positions B and D at the low end, supporting their lower overall scores. Notably, group F showed a relatively strong surface color score (18.5) but a modest overall score (82.8), implying that the limiting factor was more likely aroma rather than appearance.

The cross-section dimension was not fully aligned with the overall score. Groups A, B, D, and F scored similarly (18.1–18.4), while groups C and E were lower (15.2 and 15.1), yet their overall scores remained high (90.2 and 91.8). In Figure 2, C and E exhibit a lighter cross-section band but a high overall-score band, suggesting that cross-section was not the dominant driver of overall evaluation in this sample set, and its impact was likely outweighed by advantages in aroma and surface color under the current weighting framework. The previous literature indicates that cross-section mainly reflects internal structure and mycelial status, whereas aroma is more directly linked to flavor potential and practical value, so these indices often serve different roles in comprehensive judgment.

Based on the clustering structure in Figure 2, the six groups were classified into two major clusters. Groups A, C, and E formed one branch, sharing high surface color and aroma with overall performance above 90; although cross-section decreased in C and E, overall quality remained in a higher tier. Groups B and D formed another branch, showing low surface color and aroma, and although cross-section scores were higher, overall scores remained slightly above 80. Group F clustered closer to B and D, consistent with its low aroma score: despite relatively high surface color and cross-section, insufficient aroma limited the overall score and prevented entry into the high-score group. Overall, the clustering pattern highlighted that the upper bound of sensory quality for sauce-aroma high-temperature Daqu was primarily driven by aroma intensity and aroma cleanness, while appearance and cross-section more often determined basic qualification and structural stability, showing diminishing marginal effects on improving overall score. This also provides a clear direction for subsequent association analysis between sensory outcomes and differences in volatile components.

### 3.4. Correlation Analysis Between Sensory Scores and Key Trace Components in Sauce-Aroma High-Temperature Daqu

As shown in Table 3, trace components with VIP > 1 were not uniformly coupled with sensory dimensions. Clear directional preferences were observed. For correlation coefficients, the number of variables with an absolute value greater than 0.5 reached 31, 29, 31, and 28 in surface color, aroma, cross-section, and overall score, respectively. This pattern suggested that sensory differences in sauce-aroma high-temperature Daqu were not driven by a few single compounds. They were more likely shaped by coordinated shifts across several chemical modules. It should be noted that the sample size comprised six Daqu groups. The significance threshold for correlation tests was therefore more difficult to reach. A limited number of variables with *p* < 0.05 was consistent with statistical expectations. Under this constraint, five variables still reached significance for the overall score. Four variables were significant for aroma. Three variables were significant for surface color. Three variables were significant for cross-section. This distribution suggested that the overall score and aroma were more readily captured by volatile profiles. Cross-section and surface color were more likely external manifestations of process state and structural maturity [40,60,61,62]. Significant coupling emerged only when specific compounds stably indicated these states.

A, C, and E showed higher aroma and overall scores. B, D, and F showed lower values. This pattern was broadly consistent with the direction of significant variables. Molecules that were significantly positively associated with aroma and overall score mainly clustered in lipid oxidation-related aldehydes, high-impact sulfur compounds, and fermentative organic acids. These modules often increased brightness and diffusivity. They also enhanced the maturity notes and fermentation character. They were therefore reflected as positive contributors in the comprehensive evaluation. In contrast, molecules that were significantly negatively associated with aroma or overall score showed a stronger idiosyncratic odor background. Aromatic compounds and nitrogen-containing heterocycles with cocoa-like or medicinal tendencies could act as off-direction signals within the Daqu aroma framework of sauce-aroma high-temperature Daqu. Sensory scores were therefore reduced. Meanwhile, significant negative associations in the cross-section dimension were presented by propylene glycol, decanal, and specific pyrazines. This pattern more likely reflected tension between internal structural maturity and volatile accumulation. When certain metabolic and thermal reactions were stronger, the idealized internal-tissue state did not necessarily improve in parallel.

On this basis, 14 key trace components served as a core bridge linking chemical fingerprints to sensory differences in sauce-aroma high-temperature Daqu. They covered the most information-dense part of the correlation and significance results. They also aligned with the mechanistic narrative in which microbial metabolism and thermal reactions occurred concurrently during high-temperature stacking fermentation.

Hexanoic acid (r aroma 0.709, p 0.115; r overall score 0.801, p 0.055) showed a consistent positive trend for aroma and overall score. Statistical significance at 0.05 was not reached. Correlation strength remained high. Hexanoic acid carried a typical fatty and cheese-like background. Fermentation thickness could be directly enhanced. It could also act as an esterification precursor in subsequent brewing systems. The high correlation with overall score, therefore, suggested that high-scoring Daqu had a stronger acid supply and a more robust flavor foundation [63,64,65].

trans-3-Methyl-2-n-propylthiophane (r aroma 0.689, p 0.130; r overall score 0.794, p 0.059) also showed a positive but non-significant association. Sulfur-containing heterocycles often had very low olfactory thresholds and strong diffusion. Trace-level changes could alter mature aroma depth and complexity. In sauce-aroma systems, sulfur compounds were not required to be abundant. An appropriate window was more critical. Subtle mature, meaty, or onion-garlic accents could be provided. The Daqu aroma could be shifted from simple roasting toward a more three-dimensional sauce-aroma precursor profile. The formation mechanisms and low-threshold contributions of sulfur volatiles in Baijiu were also emphasized in existing reviews [66,67,68].

2-Hexanone oxime (r aroma 0.744, p 0.090; r overall score 0.789, p 0.062) showed a strong positive trend for aroma and overall score. Oximes were often viewed as derivative forms of carbonyls or as reaction companions. Under complex solid-state fermentation and high-temperature conditions, this compound more likely served as an indirect indicator of active carbonyl metabolism and nitrogen-related reactions. Some oximes were described as green-leafy, fresh, or slightly earthy. This orientation complemented the fresh edge required in sauce-aroma high-temperature Daqu. The positive trend, therefore, remained mechanistically plausible [69,70,71].

(E)-2-Pentenal (r aroma 0.926, p 0.008; r overall score 0.978, p 0.000744) was among the clearest positive signals for aroma and overall score. Correlation strength and significance were both high. This aldehyde was often described as green, pungent, and partly fruity. It was commonly linked to lipid oxidation or thermal reactions in food systems. Combined with the aroma advantage of high-scoring groups in the sensory heatmap, an interpretation was supported. At an appropriate level, it likely provided lifted brightness and diffusive freshness. The roasted base was therefore less dull. The overall score was consequently increased [72].

Dimethyl trisulfide (r aroma 0.866, p 0.026; r overall score 0.937, p 0.006) also showed significant positive associations. It represented a typical high-impact variable. Dimethyl trisulfide often carried cooked onion–garlic- and cabbage-like sulfur notes. The threshold was extremely low. Minor changes could reshape the aroma profile. In sauce-aroma high-temperature Daqu, this sulfur note was not necessarily expressed as a foul odor. It was more often reflected as reinforcement of mature aroma and a sauce-like background. When coexisting with pyrazines and carbonyls, roasted notes could be pushed from planar to layered. The strong positive association with the overall score, therefore, showed high mechanistic consistency [66,67,68].

Ethyl linoleate (r surface color 0.975, p 0.000925; r overall score 0.735, p 0.096) showed an extremely strong and significant positive association with surface color. It served as one of the clearest indicators of coloration in this dataset. Fatty acid ethyl esters often reflected integrated outcomes of lipid release, ethanol supply, and the esterification environment. Higher surface color scores often indicated more coordinated temperature rise and subsequent drying. Lipid-related precursor formation and esterification stability were also favored. This result suggested that surface color was not only an appearance index. It could also covary with lipid-related flavor potential [63,64].

Nonanal (r aroma 0.766, p 0.076; r overall score 0.832, p 0.040) reached a significant positive association with overall score. Nonanal often conveyed waxy and fatty notes with citrus-like and green nuances. At very low levels, it could enhance aromatic bloom. The significant positive association, therefore, suggested that high-scoring Daqu had better aroma diffusion and a cleaner fresh edge. Daqu aroma appeared brighter and cleaner. The overall evaluation was consequently improved [73,74].

Decanal (r cross-section −0.830, p 0.041; r aroma 0.740, p 0.092) showed a significant negative association with cross-section. This finding required attention. Decanal was also linked to lipid oxidation. In this dataset, a clear inverse coupling with the cross-section score was observed. When decanal increased, cross-section performance tended to decline. A more reasonable explanation was supported. Cross-section scoring emphasized compactness and uniformity. These traits could correspond to a more stable internal microenvironment and more controlled oxidation. Decanal elevation reflected more active oxidation chain reactions. Mechanistic tension, therefore, existed. This finding indicated that cross-section was not an independent index in which higher values always implied improvement. Trade-offs with parts of the volatile profile were possible [73,74,75].

4-Methylquinazoline (r surface color −0.921, p 0.009; r overall score −0.696, p 0.125) showed a significant negative association with surface color. 4-Methylquinazoline was reported to have medicinal tendencies. Its presence was often linked to nitrogen-containing heterocycle formation. A stronger heterocycle background could be formed under high-temperature reactions and nitrogen participation. In the evaluation framework of sauce-aroma high-temperature Daqu, ideal color implied maturity without overprocessing. When such heterocycles increased with stronger high-temperature reactions, reduced color scores and style deviation could occur. The significant negative association was therefore consistent [64,76].

5-Methyl-2-phenyl-2-hexenal (r aroma −0.887, p 0.018; r overall score −0.890, p 0.017) was among the most explicit negative flavor signals in this dataset. This compound was often described as cocoa-like with an aldehydic background. Within the Daqu aroma framework of sauce-aroma high-temperature Daqu, excessive cocoa-like or sweet aldehydic notes could indicate style deviation. When combined with sulfur notes and acidic notes, a less clean composite tone could form. Significant negative associations with aroma and overall score, therefore, suggested that this variable required constraint [64,77,78].

1,2,3,4-Tetramethoxybenzene (r surface color −0.998, p 0.000006; r aroma −0.602, p 0.206) showed an extremely strong and significant negative association with surface color. It was almost a reverse marker for color evaluation. Polymethoxy aromatics often conveyed sweet, woody, or medicinal tendencies. They were more likely signals derived from plant phenolic structures. This result suggested that accumulation of such aromatic ethers was linked to surface coloration deviating from the ideal state of high-scoring samples. Differences in phenolic release, thermal reaction routes, or microbial degradation could underlie this association. These differences were captured by surface color evaluation [79].

2-Ethenyl-6-methylpyrazine (r cross-section −0.866, p 0.026; r aroma 0.706, p 0.117) showed a significant negative association with cross-section. A strong positive trend was observed for aroma. Pyrazines represented a key roasted and nutty flavor framework in sauce-aroma high-temperature Daqu. They were also widely regarded as key nitrogenous odorants in Baijiu flavor research. This result suggested that enhanced pyrazine spectra improved aroma. Alignment with the ideal cross-section structure was not guaranteed. This tension matched the realistic state of high-temperature stacking fermentation. When thermal reactions and nitrogen metabolism intensified, internal structure did not necessarily shift toward a more regular aesthetic standard [69,79,80].

Acetic acid (r aroma 0.914, p 0.011; r overall score 0.871, p 0.024) was another significant positive core variable. Acetic acid directly contributed to fermentative acidity and freshness. It also acted as an esterification substrate that influenced ester formation. Significant positive associations with aroma and overall score, therefore, indicated a more active and balanced basal metabolic network in high-scoring Daqu. This signal was consistent with the general understanding that microbial metabolism dominated flavor formation in Baijiu brewing systems [63,64].

Propylene glycol (r cross-section −0.849, p 0.032; r aroma 0.515, p 0.296) showed a significant negative association with cross-section. Propylene glycol was a polyol. It was often linked to carbon flux allocation and protective metabolism under osmotic and moisture stress in microorganisms. In solid-state fermentation, it was often associated with local moisture retention and polyol accumulation. Cross-section scoring emphasized dryness, uniformity, and maturity of internal tissues. Elevated polyols could reflect stronger water retention or a shift toward polyol metabolism. An inverse relationship with cross-section evaluation was therefore plausible [64,81].

These 14 trace components covered core nodes of significant associations. They also included both positive flavor frameworks and potential negative deviation signals. For sauce-aroma high-temperature Daqu from the Chishui River Basin, this combination corresponded to a key stylistic tension. Advantages in roasted and mature notes, driven by high-temperature reactions needed to be balanced. Appropriate brightness from lipid oxidation-related aldehydes was required. Three-dimensional maturity could be enhanced by sulfur volatiles. Fermentative character and freshness were maintained by organic acids. Excessive accumulation of aromatic aldehydes and certain nitrogen heterocycles could induce style deviation. These components could therefore be defined as a core flavor set for sauce-aroma high-temperature Daqu from the Chishui River Basin. Molecular docking and molecular dynamics will be introduced in subsequent work. The binding and activation of olfactory receptor proteins by these key odorants will be explored. This approach will advance sensory differences from statistical association to mechanistic evidence at the receptor level.

### 3.5. Mechanisms by Which 14 Core Trace Components Activated the Olfactory Receptor

As shown in Table 4, the 14 core trace components exhibited a clear gradient of docking affinity and interaction modes. Acetic acid showed the strongest binding tendency (docking score = −6.655) with a highly negative electrostatic term (glide ecoul = −14.562), suggesting stabilization dominated by hydrogen bonding/polar interactions and plausibly supporting the baseline perception of fermentative acidity and freshness in Daqu aroma. In contrast, 4-methylquinazoline (−4.714) and 1,2,3,4-tetramethoxybenzene (−3.845) belonged to the high-affinity group with strongly negative van der Waals terms (glide evdw = −15.929 and −18.566), indicating hydrophobic matching and shape complementarity that could contribute medicinal/woody–warm nuances and enhance aroma texture/aftertaste. A second high-impact route involved sulfur compounds, where dimethyl trisulfide (−3.950; glide evdw = −13.611) and trans-3-methyl-2-n-propylthiophane (−3.774; glide evdw = −13.695) showed stable hydrophobically driven binding consistent with their low-threshold potential to intensify mature, onion–garlic/meaty accents and increase aroma depth; similarly, 2-ethenyl-6-methylpyrazine (−3.414; glide evdw = −13.849) supported a hydrophobic binding mode consistent with reinforcement of roasted/nutty recognition. Compounds in the moderate range were more likely to modulate aroma contour: hexanoic acid (−2.121; glide ecoul = −5.397; glide evdw = −13.009) combined polar anchoring with hydrophobic fitting, consistent with fatty/cheese-like fullness, while (E)-2-pentenal (−1.027; glide evdw = −10.705) and nonanal (−0.508; glide evdw = −14.471) plausibly contributed brightness and diffusion (green/fresh and waxy nuances). 2-hexanone oxime showed notable hydrophobic embedding (markedly negative glide evdw) and was interpreted mainly as a subtle modifier (fresh/herbal smoothing) rather than a dominant identity cue. Decanal displayed unfavorable binding (docking score = 1.952), which does not exclude sensory contribution but suggests the selected receptor model may not preferentially recognize decanal and that perception may rely on other receptor subtypes, reinforcing that sauce-aroma high-temperature Daqu aroma is encoded by multi-receptor, multi-molecule integration rather than a single-ligand pathway. A detailed interpretation is provided in File S2.

The docking results suggest that key Daqu volatiles follow a recognition logic that combines polar anchoring with hydrophobic compaction after entering the olfactory-receptor binding cavity. Polar functional groups (carboxyl, hydroxyl, oxime, and carbonyl) tend to be directionally constrained at the pocket’s polar boundary, where hydrogen-bonding and dipole stabilization are provided by polar residues (e.g., Ser75/Ser92/Ser152, Thr74/Thr76/Thr98/Thr200, Gln99, and Glu94). When needed, electrostatic or hydrogen-bond networks are reinforced by charged/polar residues (e.g., Arg150/Arg262, Asn194, His180/His261, Asp182, Gln181, and Ser258), thereby reducing ligand conformational freedom. The pocket core remains predominantly hydrophobic and is formed mainly by aliphatic residues (Leu/Ile/Val/Ala/Pro/Met), enabling continuous van der Waals contacts that govern compaction, residence time, and chain-length matching. Aromatic/heterocyclic ligands can further gain specificity through π-related contacts with aromatic residues (e.g., Phe93/Phe101/Phe102 and Tyr197), consistent with shape complementarity. Sulfur-containing ligands may exploit sulfur polarizability to form S/π interactions near aromatic residues (e.g., Phe93/Phe101/Phe102) and Met100, and may also form directional sulfur-related contacts with oxygen-containing groups of Glu94, supporting stable binding even for weakly polar ligands.

More negative docking scores generally indicate more favorable binding trends, but they are best interpreted as relative rankings within the same receptor/modeling system, rather than absolute binding affinities, due to known limitations and biases of scoring functions. Importantly, this docking analysis is computational and unvalidated experimentally in the present study, and it reflects a single-receptor, single-ligand scenario; it does not capture mixture effects (competition/antagonism) that are common in odor coding, nor does it model matrix-dependent transformations that occur during brewing. Under the canonical pathway, once polar anchoring and hydrophobic compaction support productive binding, the transmembrane bundle may undergo conformational rearrangement toward an active state, couple to Gαolf, activate adenylyl cyclase III, increase cAMP, and amplify olfactory signaling; however, receptor subtype diversity and alternative signaling routes are not addressed here. Detailed interaction maps and full residue lists are provided in File S2.

### 3.6. Microbial Community Composition Analysis of Sauce-Aroma High-Temperature Daqu

Microorganisms were major constituents of Daqu. They served as producers of hydrolytic enzymes and key contributors to characteristic flavor compounds. Therefore, microbial diversity and composition in sauce-aroma high-temperature Daqu were investigated. High-throughput sequencing was applied for profiling and subsequent analysis.

**Figure 3:**
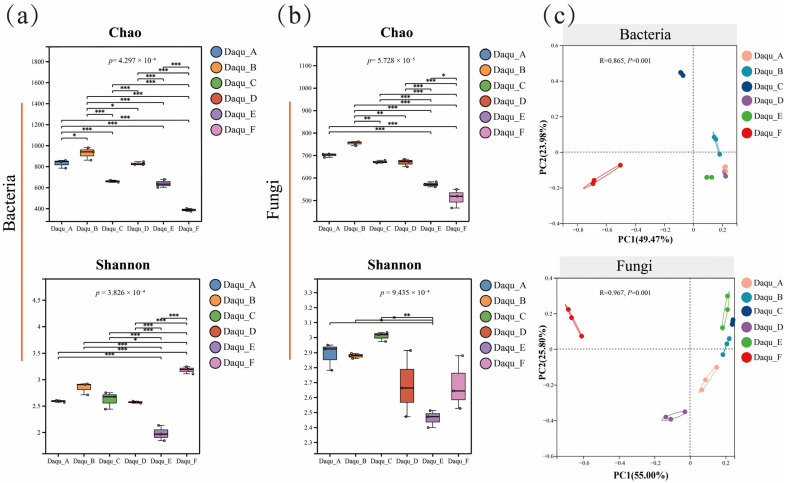

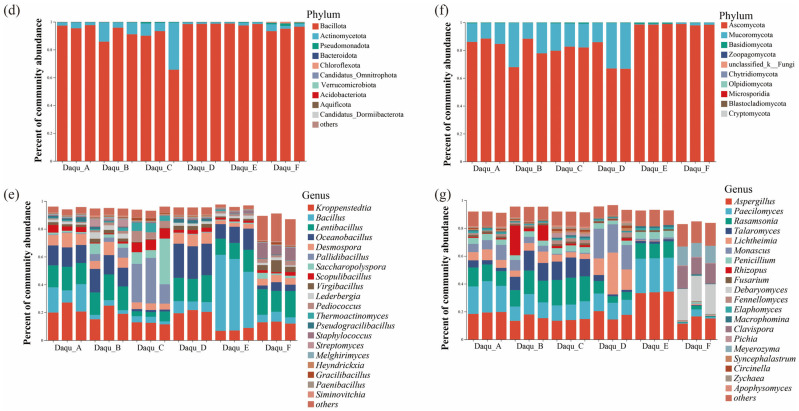
The differences in microbial diversity and structural composition within high-temperature Daqu. (**a**) Microbial diversity of bacteria; (**b**) microbial diversity of fungi; (**c**) PCoA analysis; (**d**) microbial structure composition of bacteria at the phylum level; (**e**) microbial structure composition of bacteria at the genus level; (**f**) microbial structural composition of fungi at phylum level; (**g**) microbial structural composition of fungi at genus level. Note: *p* < 0.05 (*), *p* < 0.01 (**), *p* < 0.001 (***).

In general, α-diversity was evaluated using the Chao, Shannon, and Simpson indices to assess community richness and evenness (Figure 3a,b). The results indicated that bacterial richness and diversity were higher than those of fungi. This observation was consistent with the common description of sauce-aroma high-temperature Daqu as bacterial-dominated Daqu. A plausible explanation was that the fermentation temperature exceeded 60 °C. During the high-temperature stage, most fungi were inactivated, whereas thermotolerant bacteria were retained. To determine whether microbial composition differed among samples, PCoA was used to compare community structures (Figure 3c). Significant differences were observed for both bacteria and fungi based on ANOSIM. For bacteria, r = 0.865 and *p* = 0.001. For fungi, r = 0.967 and *p* = 0.001. These compositional differences were expected to drive divergence in metabolic phenotypes across Daqu.

At the phylum level, the dominant bacterial phyla mainly comprised *Bacillota* and *Actinomycetota* (Figure 3d). The relative abundance of *Bacillota* ranged from 65.64% to 98.83%. *Actinomycetota* followed with 0.85% to 33.84%.

At the genus level, the dominant bacteria included *Kroppenstedtia*, *Bacillus*, *Lentibacillus*, *Oceanobacillus*, *Desmospora*, *Pallidibacillus*, *Saccharopolyspora*, *Scopulibacillus*, and *Virgibacillus*. This pattern was broadly consistent with earlier metagenomic surveys of sauce-aroma high-temperature Daqu.

Across all samples, *Bacillus* and *Kroppenstedtia* were the major dominant genera. *Bacillus* accounted for a substantial proportion, with a relative abundance of 2.16% to 54.56%. It was regarded as a functional cornerstone due to roles in secreting proteases and amylases, and in generating flavor precursors. *Kroppenstedtia* showed stable abundance at 6.82% to 27.15%. This genus was considered an indicator microorganism for sauce-aroma high-temperature Daqu. High prevalence was typically associated with the high-temperature fermentation environment and with the formation of pyrazine flavor compounds. Beyond these core genera, multiple thermotolerant or halotolerant genera were detected. This pattern supported the presence of a complex cooperative microbial network. *Lentibacillus*, *Oceanobacillus*, *Virgibacillus*, *Scopulibacillus*, and *Pallidibacillus* suggested natural selection under high temperature and high osmotic pressure during fermentation. The detection of *Saccharopolyspora* and *Desmospora* suggested active biosynthesis of secondary metabolites. These metabolites may have contributed to aging-related properties and characteristic aroma formation. Although genus-level relative abundances fluctuated among samples, core taxa such as *Bacillus* and *Kroppenstedtia* remained dominant. This result indicated strong directional selection imposed by the fermentation process, which supported stability and adaptability of key functional taxa. Such stability was closely linked to processing conditions, fermentation environment, and regional features.

For fungi, the dominant phyla mainly comprised *Ascomycota* and *Mucoromycota* (Figure 3f). *Ascomycota* was overwhelmingly dominant, with a relative abundance of 66.7% to 98.8%. *Mucoromycota* and *Basidiomycota* showed lower abundance and larger variation across samples. In particular, *Ascomycota* remained the leading phylum in all samples. In Daqu_A and Daqu_F, the relative abundance was 84.57% to 88.45% and 97.89% to 98.84%, respectively. As major fungal groups during Daqu fermentation, members of *Ascomycota* played important roles in organic matter degradation, fermentation participation, and synthesis of flavor components.

At the genus level, the major fungal genera included *Aspergillus*, *Paecilomyces*, *Rasamsonia*, *Talaromyces*, *Lichtheimia*, *Monascus*, and *Penicillium* (Figure 3g). Clear differences in relative abundance were observed among samples, reflecting diversity in fungal community structures. *Aspergillus* showed notable abundance, particularly in Daqu_A at 18.39% to 19.78%. *Aspergillus* was known to secrete abundant hydrolases such as amylases and proteases. These enzymes efficiently degraded starch and proteins into reducing sugars and amino acids. Such metabolites provided essential carbon and nitrogen sources for yeasts and other functional microbes. In addition, its metabolism could generate various volatiles and directly enrich the flavor profile.

Paecilomyces showed stable abundance in most samples. In Daqu_A and Daqu_E, it reached 18.76% to 22.49% and 24.36% to 24.69%, respectively. This genus contributed to organic-matter degradation and played important roles in producing 2-phenylethanol, benzaldehyde, and benzeneacetic acid ester-related compounds, and in maintaining fermentation stability. *Monascus* showed distinctive roles in Daqu. Its pigment-producing capacity and involvement in amino acid metabolism may influence flavor and color. Its metabolites also suggested potential health-related effects such as antioxidant and antimicrobial activities. *Rasamsonia* showed relatively high abundance, particularly in Daqu_B and Daqu_C at 11.79% to 16.56% and 17.99% to 19.30%. These taxa may have participated in biosynthesis of secondary metabolites during fermentation. *Talaromyces* and *Lichtheimia* were lower in abundance, indicating more limited roles in certain samples. In Daqu_F, *Lichtheimia* was 0.27% to 0.77%, indicating low activity.

Overall, strong differences were observed at the fungal genus level. Although *Aspergillus* and *Paecilomyces* were core dominant genera, their abundances varied among samples. Other genera such as *Rasamsonia* and *Talaromyces* dominated in specific samples. This pattern indicated that microbial communities during Daqu fermentation were shaped by multiple environmental factors and showed strong regional and process-dependent features.

### 3.7. Correlations Between Dominant Bacterial and Fungal Genera and 14 Key Compounds in Sauce-Aroma High-Temperature Daqu Samples

Correlations between dominant bacterial and fungal genera and the 14 key compounds were analyzed to clarify the complex links between microbial communities and flavor and aroma compounds in Daqu fermentation. The results indicated that bacteria and fungi not only produced specific compounds through metabolism, but also shaped flavor formation through interspecies interactions during fermentation.

**Figure 4:**
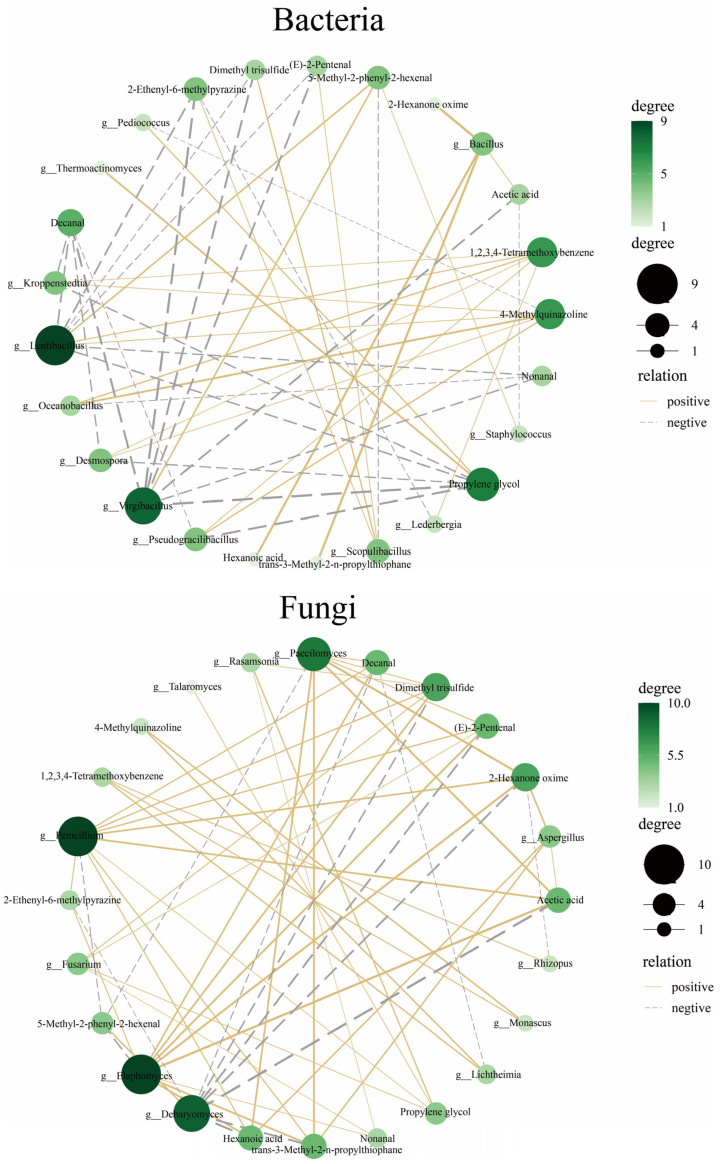
Correlation analysis network diagrams: The correlation network between dominant microbials and compounds of Daqu. The size of the circles is linked to the number of the edge of compounds. The thickness of lines is proportional to the value of spearman’s correlation (|ρ| > 0.6, *p* < 0.05).

As shown in Figure 4, the correlation analysis suggested that *Aspergillus*, *Paecilomyces*, *Monascus*, *Bacillus*, *Kroppenstedtia*, and *Lentibacillus* exerted marked influences on flavor generation during Daqu fermentation. In the bacterial analysis, *Bacillus* exhibited significant positive correlations with several key compounds, particularly with acetic acid. This pattern indicated that metabolically active *Bacillus* might have promoted acetic acid formation. As an important organic acid in Daqu fermentation, acetic acid directly affected sourness and flavor complexity. In addition, a positive association between *Bacillus* and 1,2,3,4-tetramethoxybenzene suggested that this genus might have facilitated the formation of aromatic compounds, thereby enhancing the layering of Daqu aroma. In contrast, *Lentibacillus* showed negative correlations with multiple flavor compounds such as (E)-2-pentenal and dimethyl trisulfide. This negative relationship implied that *Lentibacillus* might have suppressed the formation of these compounds via metabolic regulation, thereby influencing overall flavor.

In the fungal analysis, *Monascus* showed a significant positive correlation with acetic acid. This result suggested that *Monascus* might have promoted acetic acid formation through its metabolic activity, which strengthened sourness and increased aroma intensity. A positive association between *Monascus* and 1,2,3,4-tetramethoxybenzene also suggested that this fungus might have enhanced the generation of certain aromatic compounds, adding complex aromatic layers to the flavor profile. By contrast, *Paecilomyces* and *Penicillium* exhibited negative correlations with flavor compounds such as 5-methyl-2-phenyl-2-hexenal and decanal. This pattern indicated that these fungi might have inhibited the formation of these compounds through metabolism, thereby modulating aroma characteristics [82].

Members of the genus *Virgibacillus* are ubiquitous bacteria in Daqu, with previous surveys highlighting them as dominant taxa in high-temperature Daqu (HTD). Specifically, research into the microecological divergence of black, yellow, and white Daqu indicated a markedly higher relative abundance of *Virgibacillus* in yellow Daqu compared to the other two types. While the functional contributions of *Virgibacillus* to fermentation are not yet fully elucidated, initial findings suggest it is among six “chameleon microorganisms” that play a fundamental role in the microecological differentiation of HTD [14,83,84].

The analysis of positively correlated microorganisms indicated that *Bacillus* and *Monascus* played promoting roles in flavor formation. *Bacillus* appeared to enhance sourness and enrich flavor layering by promoting acetic acid formation. *Monascus* appeared to deepen sourness and aroma by promoting the formation of acetic acid and aromatic compounds such as 1,2,3,4-tetramethoxybenzene. These metabolic activities contributed substantially to sourness, aroma, and overall flavor complexity. Although *Paecilomyces* and *Penicillium* showed negative correlations with certain flavor compounds, this pattern did not indicate irrelevance. Instead, negatively correlated genera such as *Paecilomyces* and *Penicillium* might have regulated flavor balance by constraining the synthesis of specific compounds. Even with inhibitory tendencies, these fungi still contributed to shaping the overall aroma and flavor characteristics [85].

Significantly, in open, spontaneous Daqu manufacture, environmental microorganisms can be introduced transiently, including taxa that are often treated as spoilage-associated indicators in fermented foods (e.g., members of *Enterobacteriaceae*). However, sauce-aroma high-temperature Daqu undergoes a pronounced high-temperature stage (commonly reaching 60–70 °C) together with progressive dehydration/low moisture, which collectively imposes a strong selective pressure that favors thermotolerant functional groups (e.g., *Bacillus* and other heat-adapted taxa) while suppressing the outgrowth of many mesophilic, spoilage-associated organisms [86,87,88]. Although heat-resistant *Enterobacteriaceae* variants can still be detected in some Daqu fermentations (indicating that high temperature is not an absolute sterilization step), their persistence is typically constrained by the combined effects of thermal stress, low water activity, and competitive starter ecology [89]. In our metagenomic profiles of mature (post-maturation) Daqu, spoilage-associated bacterial groups (e.g., *Enterobacteriaceae*) were nearly undetectable, which we interpret as a “clean” microbial signature and a practical quality indicator: it suggests that the starter has passed through the intended high-temperature selective barrier and reached a stable ecological state more likely to support consistent enzymatic functionality and reduce the likelihood of atypical fermentation trajectories or off-note risk in subsequent brewing [24,82,90,91].

Overall, correlation analysis between dominant bacterial and fungal genera and 14 key compounds revealed a complex relationship between microbial ecology and flavor and aroma compounds. The results indicated that bacteria and fungi not only produced specific compounds, but also influenced flavor formation through interactions within the community. On this basis, the following key microorganisms showing significant influences during fermentation were identified. (1) *Aspergillus* played an important role in fermentation and significantly affected flavor characteristics by promoting the formation of sourness- and aroma-related compounds. (2) *Paecilomyces* regulated flavor balance through negative correlations with multiple flavor compounds and served an important modulatory role. (3) *Monascus* positively contributed to the formation of acetic acid and aromatic compounds, thereby enhancing sourness and deepening aroma. (4) *Bacillus* contributed substantially to sourness and flavor complexity by promoting acetic acid formation. (5) *Kroppenstedtia* and *Lentibacillus* influenced flavor compound synthesis through metabolic activity and modulated flavor layering.

### 3.8. Discussion

A growing body of Baijiu and Daqu research has moved from single-layer profiling toward integrated flavoromics, yet many studies still treat volatile fingerprints, sensory outcomes, and process drivers as loosely connected endpoints. Against this backdrop, the present work positions sauce-aroma high-temperature Daqu quality as a stratified chemical phenotype, where a 210-compound headspace volatile matrix separates into abundance tiers and highlights pyrazines as a dominant signature. This aligns with reports that high-temperature Daqu tends to be enriched in Maillard-derived nitrogen heterocycles, with tetramethylpyrazine repeatedly recognized as a hallmark contributor to roasted and nutty impressions in sauce-flavor systems [92,93]. At the same time, the emphasis on aldehydes and sulfur volatiles as quality-associated variables is consistent with broader sauce-flavor Baijiu literature showing that carbonyls and trace sulfur odorants, despite low concentrations, can exert disproportionate sensory leverage because of low thresholds and high diffusivity [3,35].

The microbial results also resonate with well-established ecological principles of Daqu fermentation, where bio-heat and dehydration create strong selection that favors thermotolerant bacterial consortia and reduces fungal breadth, producing community trajectories that are reproducible across regions and process variants [94,95]. Dominance of *Bacillus* in high-temperature Daqu has been widely reported and is typically interpreted through its enzyme secretion capacity and its role in generating precursor pools that feed downstream aroma formation, while *Kroppenstedtia* and related thermophiles are often discussed as environment-adapted taxa associated with high-temperature niches and characteristic flavor directionality in sauce-flavor production chains [94,95,96]. What this study adds relative to many sequencing surveys is the explicit linkage between dominant genera and discriminant odorants, which parallels newer work that couples microbiome shifts to volatile outputs during Daqu or heap fermentation but usually stops at correlation and pathway inference [93,97,98]. A critical reading suggests that the reported genus compound associations are best viewed as hypotheses about metabolic routing and community-level division of labor, and future causal confirmation would benefit from time-resolved multi-omics, isolate-based reconstructions, or controlled consortia fermentations that can separate production from consumption and adsorption effects [93,97].

The most distinctive scientific move is the attempt to bridge statistical discrimination and sensory coupling with receptor-level plausibility, shifting the discussion from what correlates with quality to what could be physically recognized by an odor receptor pocket under realistic chemical constraints. Traditional aroma studies in Daqu frequently rely on HS-SPME GC MS combined with GC olfactometry and OAV-based prioritization, which is powerful for identifying odor-active molecules but does not directly test whether a proposed marker set is compatible with receptor-binding logic [69]. In contrast, docking-guided interpretation follows an emerging computational chemical-senses literature where structure-based models, dynamics, and virtual screening are used to rationalize ligand recognition and to propose testable residue-level hypotheses, while also acknowledging that odor coding is multi-receptor and mixture-dependent [99,100,101]. Taken together, this study is scientifically unique because it connects chemical fingerprints, sensory relevance, microbial ecology, and receptor-scale mechanisms into a single quality control narrative, yielding a compact set of actionable targets that are not only statistically discriminant but also mechanistically interpretable and, therefore, more transferable across batches, sites, and process adjustments [97,99].

## 4. Limitations and Prospects

This study was designed as a focused, proof-of-concept mapping between trace volatiles, sensory performance, and microbial ecology, yet its statistical inferences should be interpreted as hypothesis-generating, because the dataset contains only six independent Daqu groups and the overall sensory scores are concentrated in a relatively high band from 80.6 to 93.5 on a 100-point scale. Looking ahead, expanding the sampling space across production sites, seasons, batches, and process windows is the most direct way to improve power and transportability, which is consistent with broader evidence that Daqu microbiota and flavor phenotypes are strongly temperature- and process-driven and can vary systematically under different incubation trajectories.

Because the commercial target is marketable Baijiu, an important next step is to connect Daqu markers to the sensory and chemical outcomes of the liquor itself rather than relying on Daqu sensory outcomes alone, especially since many volatiles can be lost during fermentation and distillation while additional compounds can be induced or amplified across the saccharification, fermentation, and post-distillation stages. A staged validation design could track the same batches from Daqu to fermented grains to base Baijiu and, when possible, to aged liquor, testing whether the proposed discriminant set predicts downstream aroma quality and whether any markers function as stable proxies or only as early process indicators. Embedding sensory methods that mirror distillery practice would further strengthen interpretability, since industry scoring systems integrate cues that reflect production realities, and aligning laboratory panels with those criteria helps ensure that chemical signatures are evaluated against a commercially meaningful definition of quality.

The current scope also emphasizes headspace volatiles and amplicon profiling, so future work can broaden the mechanistic foundation by adding affordable but informative measurements, such as proximate composition, acidity and pH, soluble carbohydrates, residual starch, nonvolatile fermentation products, and especially enzyme activities that are widely recognized as critical functional outputs of Daqu microbiota. In parallel, Daqu is a dynamic culture with succession over time, so time-resolved sampling across incubation and maturation would help separate transient blooms from stable functional states, an issue repeatedly highlighted in high-temperature Daqu succession studies. Finally, the olfactory receptor modeling can be framed as a mechanistic plausibility layer that prioritizes ligands and residues for targeted validation rather than as a standalone proof of odor coding, since docking scores require experimental corroboration and odor perception is shaped by multi-receptor, mixture-dependent interactions. Future directions that fit naturally with the present framework include testing a small receptor panel with heterologous expression, using mutagenesis to probe key polar residues, and applying mixture models or competitive binding concepts to evaluate non-additivity in complex matrices.

## 5. Conclusions

In this study, volatile profiles and microbial communities were jointly characterized in six groups of sauce-aroma high-temperature Daqu. A total of 210 volatile trace compounds were quantified by HS-SPME-GC-MS. Fourteen chemical classes showed significant stratification across samples. The high-abundance group was dominated by pyrazines, and the separation was largely driven by tetramethylpyrazine and 2,3,5-trimethylpyrazine. OPLS-DA and VIP indicated that discriminatory signals originated from pyrazine derivatives, lipid oxidation-related aldehydes and ketones, acids and their ethyl esters, sulfur-containing compounds, and selected aromatic compounds. In sensory evaluation, aroma best explained the overall score. (E)-2-pentenal and dimethyl trisulfide were significantly and positively correlated with aroma and overall score. Receptor docking suggested that polar small molecules and hydrophobic heterocycles jointly contributed to olfactory recognition. Sequencing results showed higher bacterial diversity than fungal diversity. Dominant bacterial genera were *Bacillus*, *Kroppenstedtia*, and *Lentibacillus*. Dominant fungal genera included *Aspergillus*, *Paecilomyces*, *Monascus*, and *Penicillium*. Correlation patterns suggested that *Bacillus* and *Monascus* were more likely to promote the accumulation of acetic acid and aromatic compounds.

By integrating chemical fingerprints, sensory coupling, and microbial evidence, the core trace compounds were identified as acetic acid, hexanoic acid, (E)-2-pentenal, nonanal, decanal, dimethyl trisulfide, trans-3-methyl-2-n-propylthiophane, 2-hexanone oxime, ethyl linoleate, propylene glycol, 2-ethenyl-6-methylpyrazine, 4-methylquinazoline, 5-methyl-2-phenyl-2-hexenal, and 1,2,3,4-tetramethoxybenzene. The core microorganisms were identified as *Bacillus*, *Kroppenstedtia*, *Lentibacillus*, *Aspergillus*, *Paecilomyces*, *Monascus*, and *Penicillium*.

## Figures and Tables

**Table 1:** Sensory dimension scoring criteria for sauce-aroma high-temperature Daqu.

Sensory Dimension	Score	Evaluation Focus	Scoring Criteria
Surface color	20 points	Overall surface color and uniformity, degree of yellow-brown or dark-brown, proportion of gray-white, abnormal mold bloom and cracks	Premium grade: Surface was mainly yellow-brown or dark-brown, 17–20 points. Good grade: Surface was largely yellow-brown or dark-brown with only a small amount of gray-white, 14–16 points. Standard grade: Surface appeared gray-white or showed a high proportion of gray-white, 12–13 points. Further deductions were applied when obvious abnormal mold spots or abnormal dampness were present.
Aroma	60 points	Clarity and harmony of sauce aroma, Daqu aroma, douchi aroma, or toasted aroma; intensity of composite aroma; off-odor and moldy spoilage cues	Premium grade: Sauce aroma, Daqu aroma, and douchi aroma were prominent, and composite aroma was evident, 54–60 points. Good grade: Sauce aroma, Daqu aroma, and toasted aroma were relatively clear, and composite aroma was distinct, 42–53 points. Standard grade: Daqu aroma and sauce aroma were present, But composite aroma was weak, and minor off-odors could occur, 36–41 points. Samples with obvious off-odors were downgraded further.
Cross-section	20 points	Consistency of cross-section color, gray-white or gray-yellow appearance, uniformity of pores and structure, extent of mycelial appearance, dryness of the core	Premium grade: Cross-section color was consistent, mycelia were obvious, and the core was relatively dry, 17–20 points. Good grade: Cross-section was relatively consistent, mycelia were fairly obvious, and the core contained a small amount of moisture, 14–16 points. Standard grade: Cross-section consistency was moderate, and contaminating microbes could be observed or the core moisture was high, 12–13 points. Further deductions were applied when contamination and a wet core were evident.
Overall grade	100	Overall sensory quality score	Premium grade: Total score was greater than 90 points. Good grade: Total score was 70–90 points. Standard grade: Total score was 60–70 points. Samples below 60 points were considered unqualified.

**Table 2:** VIP values of trace components in different sauce-aroma high-temperature Daqu samples (>1.000).

Number	Compound Name	VIP
1	2-Pentanol	1.263
2	Butyl acrylate	1.260
3	(Z)-Oxacyclopentadec-6-en-2-one	1.241
4	γ-Linolenic acid	1.232
5	5-Butyl-4-methyl-2(3H)-furanone	1.226
6	Hexanoic acid	1.210
7	trans-3-Methyl-2-n-propylthiophane	1.190
8	Ethyl octanoate	1.184
9	Benzoic acid	1.178
10	1-Pentanol	1.176
11	4-Octanone	1.176
12	2-Pentylfuran	1.174
13	Ethyl 2-methylbutanoate	1.174
14	1,2,3,4,5-Pentamethoxybenzene	1.173
15	3-Octanol	1.173
16	1,4-Dimethoxybenzene	1.172
17	Ethyl 4-methylpentanoate	1.171
18	2-Hexanone oxime	1.161
19	(E)-2-Pentenal	1.149
20	(E)-2-Nonenal	1.143
21	Dimethyl trisulfide	1.142
22	Cyclohept-4-en-1-ol	1.136
23	Methyl 3,4-dimethylbenzoate	1.133
24	2-Methylpropanal	1.109
25	β-Methylcinnamaldehyde	1.109
26	2-Ethenyl-6-methylpyrazine	1.105
27	2-Propanamine	1.105
28	Allyl acetate	1.104
29	Acetic acid	1.098
30	Propylene glycol	1.097
31	Octanal	1.095
32	4,5-Dihydro-5,5,7-trimethyl-6H-[1,2,5] oxadiazolo[3,4-b] [1,4] diazepine	1.092
33	2-Hydroxy-3-pentanone	1.091
34	Benzyl methyl sulfide	1.089
35	1,2,3-Trimethoxybenzene	1.088
36	1,3-Hexadienylbenzene	1.087
37	Octyloxirane	1.085
38	2-Phenyl-1H-imidazole	1.085
39	2-Phenylethyl nicotinate	1.084
40	Ethyl 2-methylpropanoate	1.084
41	2-Dodecanone	1.083
42	2,3,5-Trimethyl-6-propylpyrazine	1.082
43	2-Nonanone	1.082
44	2-Methyl-1-propanol	1.080
45	Methyl linoleate	1.080
46	7-Oxooctanoic acid	1.075
47	Propionic acid	1.075
48	Ethyl 4-hydroxybenzoate	1.072
49	Methyl 6-nonynoate	1.069
50	N, N-Dimethylmethylamine	1.069
51	4-Methylhexanoic acid	1.067
52	Angelic acid	1.064
53	Cyclotridecanone	1.061
54	Methyl anthranilate	1.060
55	Hexanal	1.058
56	Menthol	1.057
57	Decyloctyl ether	1.056
58	Ethyl pentadecanoate	1.055
59	Acetaldehyde	1.051
60	Octanoic acid	1.051
61	2-Methylpropanoic acid	1.046
62	(Z)-Undec-6-en-2-one	1.040
63	Ethyl valerate	1.039
64	Ethyl oleate	1.039
65	Ethyl linoleate	1.038
66	Ethyl butyrate	1.035
67	(E)-2-Methyl-2-pentenoic acid	1.035
68	2-Phenylethyl 2-methylpropanoate	1.031
69	1-Pentadecene	1.030
70	Nonanal	1.028
71	3-Methylbutanoic acid	1.025
72	Nonanoic acid	1.024
73	3-Methyl-1-butanol	1.024
74	3,7-Dimethyl-1,6-octadien-3-yl formate	1.024
75	2-Octanone	1.024
76	4-Methylquinazoline	1.022
77	3-Methyl-2,5-piperazinedione	1.021
78	(Z)-Benzaldehyde oxime	1.016
79	Decanal	1.015
80	Ethyl acetate	1.014
81	2-Methylhexanoic acid	1.013
82	Acetone	1.012
83	N-(2-Methylpropyl) acetamide	1.012
84	3-Methyltridecane	1.012
85	2-Acetyl-3,4,6-trimethylpyrazine	1.010
86	2-(Phenylpiperidin-1-ylmethyl) cyclohexanol	1.007
87	5-Methyl-2-phenyl-2-hexenal	1.007
88	1,2,3,4-Tetramethoxybenzene	1.003
89	Methylpyrazine	1.003
90	2,3-Butanedione	1.000

**Table 3:** Correlation analysis between sensory scores and key trace components in sauce-aroma high-temperature Daqu.

Compound Name	r: Surface Color	r: Aroma	r: Cross-Section	r: Overall Score	*p*: Surface Color	*p*: Aroma	*p*: Cross- Section	*p*: Overall Score
2-Pentanol	0.357	0.404	0.263	0.577	0.488	0.427	0.615	0.230
Butyl acrylate	0.357	0.404	0.263	0.577	0.488	0.427	0.615	0.230
(Z)-Oxacyclopentadec-6-en-2-one	0.357	0.404	0.263	0.577	0.488	0.427	0.615	0.230
γ-Linolenic acid	0.357	0.404	0.263	0.577	0.488	0.427	0.615	0.230
5-Butyl-4-methyl-2(3H)-furanone	0.238	0.538	−0.118	0.553	0.650	0.271	0.824	0.255
**Hexanoic acid**	**0.537**	**0.709**	**−0.181**	**0.801**	**0.272**	**0.115**	**0.732**	**0.055**
**trans-3-Methyl-2-n-propylthiophane**	**0.528**	**0.689**	**−0.131**	**0.794**	**0.281**	**0.130**	**0.805**	**0.059**
Ethyl octanoate	0.134	0.314	0.230	0.404	0.801	0.544	0.662	0.427
Benzoic acid	0.310	0.386	−0.616	0.298	0.550	0.450	0.193	0.566
1-Pentanol	0.310	0.386	−0.616	0.298	0.550	0.450	0.193	0.566
4-Octanone	0.642	0.346	0.167	0.599	0.169	0.502	0.752	0.209
2-Pentylfuran	0.416	0.158	−0.471	0.165	0.412	0.765	0.345	0.755
Ethyl 2-methylbutanoate	0.310	0.386	−0.616	0.298	0.550	0.450	0.193	0.566
1,2,3,4,5-Pentamethoxybenzene	0.310	0.386	−0.616	0.298	0.550	0.450	0.193	0.566
3-Octanol	−0.032	−0.183	0.742	0.025	0.952	0.728	0.091	0.963
1,4-Dimethoxybenzene	0.310	0.386	−0.616	0.298	0.550	0.450	0.193	0.566
Ethyl 4-methylpentanoate	0.310	0.386	−0.616	0.298	0.550	0.450	0.193	0.566
**2-Hexanone oxime**	**0.542**	**0.744**	**−0.348**	**0.789**	**0.266**	**0.090**	**0.500**	**0.062**
**(E)-2-Pentenal**	**0.717**	**0.926**	**−0.504**	**0.978**	**0.109**	**0.008**	**0.308**	**0.001**
(E)-2-Nonenal	0.399	0.429	0.195	0.597	0.433	0.396	0.712	0.211
**Dimethyl trisulfide**	**0.685**	**0.866**	**−0.409**	**0.937**	**0.133**	**0.026**	**0.421**	**0.006**
Cyclohept-4-en-1-ol	−0.497	−0.162	0.028	−0.321	0.316	0.759	0.958	0.534
Methyl 3,4-dimethylbenzoate	0.310	0.386	−0.616	0.298	0.550	0.450	0.193	0.566
2-Methylpropanal	0.453	−0.105	−0.239	−0.002	0.367	0.843	0.648	0.997
β-Methylcinnamaldehyde	0.427	0.519	−0.541	0.485	0.399	0.291	0.267	0.329
**2-Ethenyl-6-methylpyrazine**	**0.373**	**0.706**	**−0.866**	**0.548**	**0.467**	**0.117**	**0.026**	**0.260**
2-Propanamine	−0.302	−0.129	0.483	−0.093	0.561	0.808	0.332	0.860
Allyl acetate	−0.227	0.049	0.463	0.093	0.665	0.926	0.356	0.860
**Acetic acid**	**0.433**	**0.914**	**−0.483**	**0.871**	**0.391**	**0.011**	**0.332**	**0.024**
**Propylene glycol**	**0.194**	**0.515**	**−0.849**	**0.312**	**0.713**	**0.296**	**0.032**	**0.548**
Octanal	0.528	0.624	−0.242	0.702	0.282	0.185	0.644	0.120
4,5-Dihydro-5,5,7-trimethyl-6H-[1,2,5] oxadiazolo[3,4-b] [1,4] diazepine	−0.654	−0.350	0.323	−0.470	0.159	0.497	0.532	0.347
2-Hydroxy-3-pentanone	0.710	0.278	−0.012	0.510	0.114	0.593	0.983	0.301
Benzyl methyl sulfide	−0.654	−0.350	0.323	−0.470	0.159	0.497	0.532	0.347
1,2,3-Trimethoxybenzene	0.413	0.317	−0.541	0.292	0.416	0.540	0.268	0.574
1,3-Hexadienylbenzene	−0.654	−0.350	0.323	−0.470	0.159	0.497	0.532	0.347
Octyloxirane	−0.654	−0.350	0.323	−0.470	0.159	0.497	0.532	0.347
2-Phenyl-1H-imidazole	−0.033	0.312	−0.180	0.228	0.950	0.547	0.732	0.664
2-Phenylethyl nicotinate	−0.654	−0.350	0.323	−0.470	0.159	0.497	0.532	0.347
Ethyl 2-methylpropanoate	−0.654	−0.350	0.323	−0.470	0.159	0.497	0.532	0.347
2-Dodecanone	−0.654	−0.350	0.323	−0.470	0.159	0.497	0.532	0.347
2,3,5-Trimethyl-6-propylpyrazine	−0.165	0.094	0.456	0.155	0.754	0.859	0.363	0.769
2-Nonanone	−0.699	−0.301	0.559	−0.375	0.122	0.562	0.249	0.463
2-Methyl-1-propanol	−0.248	−0.677	0.316	−0.631	0.635	0.140	0.542	0.180
Methyl linoleate	0.222	−0.361	0.662	−0.071	0.672	0.482	0.152	0.893
7-Oxooctanoic acid	−0.418	−0.102	0.454	−0.119	0.410	0.847	0.366	0.823
Propionic acid	0.101	0.142	0.346	0.264	0.849	0.789	0.502	0.613
Ethyl 4-hydroxybenzoate	−0.654	−0.350	0.323	−0.470	0.159	0.497	0.532	0.347
Methyl 6-nonynoate	−0.654	−0.350	0.323	−0.470	0.159	0.497	0.532	0.347
N, N-Dimethylmethylamine	0.149	−0.054	−0.352	−0.095	0.778	0.919	0.494	0.858
4-Methylhexanoic acid	−0.634	−0.276	0.594	−0.319	0.176	0.597	0.214	0.538
Angelic acid	0.491	−0.109	0.486	0.210	0.323	0.837	0.329	0.690
Cyclotridecanone	−0.536	−0.212	0.418	−0.273	0.273	0.687	0.409	0.601
Methyl anthranilate	−0.020	0.155	−0.424	0.019	0.970	0.769	0.402	0.971
Hexanal	−0.239	0.300	0.163	0.238	0.648	0.564	0.757	0.649
Menthol	0.493	−0.074	0.481	0.242	0.321	0.889	0.334	0.644
Decyloctyl ether	0.592	0.514	−0.431	0.571	0.216	0.296	0.394	0.237
Ethyl pentadecanoate	0.472	0.690	−0.546	0.659	0.344	0.129	0.263	0.155
Acetaldehyde	0.556	−0.116	−0.229	0.028	0.251	0.827	0.663	0.957
Octanoic acid	0.412	0.179	0.213	0.374	0.417	0.734	0.685	0.465
2-Methylpropanoic acid	0.097	0.798	−0.434	0.655	0.856	0.057	0.390	0.158
(Z)-Undec-6-en-2-one	−0.595	−0.272	0.387	−0.358	0.213	0.603	0.448	0.486
Ethyl valerate	0.446	0.308	0.106	0.476	0.376	0.552	0.841	0.340
Ethyl oleate	0.202	−0.291	0.575	−0.037	0.701	0.576	0.232	0.944
**Ethyl linoleate**	**0.975**	**0.511**	**−0.325**	**0.735**	**0.001**	**0.300**	**0.530**	**0.096**
Ethyl butyrate	0.462	0.690	−0.724	0.606	0.356	0.129	0.104	0.202
(E)-2-Methyl-2-pentenoic acid	0.465	−0.085	−0.172	0.040	0.353	0.873	0.744	0.940
2-Phenylethyl 2-methylpropanoate	0.451	0.674	−0.677	0.600	0.370	0.142	0.140	0.208
1-Pentadecene	−0.418	−0.206	0.444	−0.218	0.409	0.696	0.378	0.679
**Nonanal**	**0.807**	**0.766**	**−0.608**	**0.832**	**0.052**	**0.076**	**0.201**	**0.040**
3-Methylbutanoic acid	−0.179	0.381	0.049	0.304	0.735	0.456	0.926	0.558
Nonanoic acid	−0.334	0.011	0.197	−0.054	0.518	0.983	0.708	0.918
3-Methyl-1-butanol	0.060	−0.445	0.101	−0.364	0.910	0.376	0.850	0.478
3,7-Dimethyl-1,6-octadien-3-yl formate	−0.251	−0.052	0.465	−0.009	0.632	0.922	0.353	0.986
2-Octanone	0.072	0.411	−0.659	0.225	0.892	0.418	0.154	0.669
**4-Methylquinazoline**	**−0.921**	**−0.532**	**0.465**	**−0.696**	**0.009**	**0.277**	**0.353**	**0.125**
3-Methyl-2,5-piperazinedione	0.333	0.531	−0.646	0.433	0.519	0.278	0.166	0.390
(Z)-Benzaldehyde oxime	0.333	0.531	−0.646	0.433	0.519	0.278	0.166	0.390
**Decanal**	**0.501**	**0.740**	**−0.830**	**0.637**	**0.311**	**0.092**	**0.041**	**0.174**
Ethyl acetate	0.555	0.397	−0.587	0.405	0.253	0.436	0.221	0.426
2-Methylhexanoic acid	0.367	−0.396	0.021	−0.230	0.474	0.437	0.969	0.661
Acetone	0.333	0.531	−0.646	0.433	0.519	0.278	0.166	0.390
N-(2-Methylpropyl) acetamide	0.333	0.531	−0.646	0.433	0.519	0.278	0.166	0.390
3-Methyltridecane	−0.062	0.239	−0.400	0.088	0.906	0.648	0.432	0.868
2-Acetyl-3,4,6-trimethylpyrazine	0.333	0.531	−0.646	0.433	0.519	0.278	0.166	0.390
2-(Phenylpiperidin-1-ylmethyl) cyclohexanol	0.333	0.531	−0.646	0.433	0.519	0.278	0.166	0.390
**5-Methyl-2-phenyl-2-hexenal**	**−0.511**	**−0.887**	**0.423**	**−0.890**	**0.301**	**0.018**	**0.403**	**0.017**
**1,2,3,4-Tetramethoxybenzene**	**−0.998**	**−0.602**	**0.511**	**−0.776**	**0.000**	**0.206**	**0.300**	**0.070**
Methylpyrazine	0.191	−0.173	−0.035	−0.102	0.717	0.743	0.948	0.848
2,3-Butanedione	0.695	0.241	−0.007	0.472	0.126	0.646	0.990	0.345

Note: r: Surface Color, r: Aroma, r: Cross-section, and r: Overall Score represented the correlations between each trace component and the corresponding sensory dimension score. p: Surface Color, p: Aroma, p: Cross-section, and p: Overall Score represented the significance of the correlations between each trace component and the corresponding sensory dimension score. The compounds shown in bold and with blue shading in the table are the core compounds identified after screening of Daqu and are highlighted accordingly.

**Table 4:** Docking scores of 14 core trace components for activation of the olfactory receptor.

Compound Name	Docking Score	Glide Ecoul	Glide Emodel	Glide Evdw
Acetic acid	−6.655	−14.562	−41.101	−4.830
4-Methylquinazoline	−4.714	−0.531	−21.129	−15.929
5-Methyl-2-phenyl-2-hexenal	−4.578	−0.318	−20.426	−17.022
Propylene glycol	−4.022	−10.972	−21.636	−8.251
Dimethyl trisulfide	−3.950	−0.414	−16.924	−13.611
1,2,3,4-Tetramethoxybenzene	−3.845	−0.720	−21.225	−18.566
trans-3-Methyl-2-n-propylthiophane	−3.774	−0.534	−15.895	−13.695
2-Ethenyl-6-methylpyrazine	−3.414	−1.282	−17.958	−13.849
Hexanoic acid	−2.121	−5.397	−18.345	−13.009
2-Hexanone oxime	−1.666	−0.736	−16.098	−14.212
(E)-2-Pentenal	−1.027	−0.136	−9.989	−10.705
2-Hexanone oxime	−0.871	−0.726	−13.351	−12.092
Nonanal	−0.508	−0.645	−13.211	−14.471
Decanal	1.952	−0.735	−14.879	−17.428

Note: Docking Score was the primary metric for ligand ranking and selection. Lower values indicated better evaluation. Units were kcal/mol (same below). Glide Ecoul was the Coulombic (electrostatic) interaction energy between the receptor and ligand. Glide Emodel was used for pose selection and stability comparison within the same ligand. It was mainly applied to rank and select among different poses of the same ligand. Glide Evdw was the van der Waals interaction energy between the receptor and ligand derived from the force field vdW term.

## Data Availability

The original contributions presented in this study are included in the article/Supplementary Materials. Further inquiries can be directed to the corresponding author.

## Supplementary Materials

The following supporting information can be downloaded at: https://www.mdpi.com/article/10.3390/foods15030599/s1. File S1. OPLS-DA of different sauce-aroma high-temperature Daqu samples (a) and model cross-validation results (b). File S2. Mechanisms by which 14 core trace components activated the olfactory receptor. Figure S1. These photos show the olfactory receptor (a) and its microscopic molecular interaction forces (b). The schematic diagrams of interactions between the olfactory receptor and the following compounds are provided in subfigures (c) to (p), respectively: Hexanoic acid, trans-3-Methyl-2-n-propylthiophane, 2-Hexanone oxime, (E)-2-Pentenal, Dimethyl trisulfide, Ethyl linoleate, Nonanal, Decanal, 4-Methylquinazoline, 5-Methyl-2-phenyl-2-hexenal, 1,2,3,4-Tetramethoxybenzene, 2-Ethenyl-6-methylpyrazine, Acetic acid, and Propylene glycol. Table S1. Concentrations of volatile components in high-temperature Daqu. Table S2. Detailed sensory evaluation dataset for sauce-aroma high-temperature Daqu.

## Abbreviations

The following abbreviations are used in this manuscript:

AC III	Adenylyl cyclase III
ANOSIM	Analysis of similarities
bp	base pairs
cAMP	cyclic adenosine monophosphate
CD-HIT	Cluster Database at High Identity with Tolerance
Ecoul	Coulomb (electrostatic) interaction energy term
EI	electron ionization
Emodel	Glide Emodel
Evdw	van der Waals interaction energy term
GC-MS	gas chromatography–mass spectrometry
Gαolf	alpha subunit of the olfactory G protein, G(olf)
HS-SPME-GC-MS	headspace solid-phase microextraction–gas chromatography–mass spectrometry
MEGAHIT	MEGAHIT (metagenome assembly program name)
miniGs	engineered mini Gs protein
*m*/*z*	mass-to-charge ratio
NIST	National Institute of Standards and Technology
OPLS-DA	orthogonal partial least squares discriminant analysis
OPLS4	Optimized Potentials for Liquid Simulations 4
ORF/ORFs	open reading frame(s)
OR51E2	olfactory receptor family 51 subfamily E member 2
PCoA	principal coordinates analysis
RCSB PDB	Research Collaboratory for Structural Bioinformatics Protein Data Bank
RMSD	root mean square deviation
SP (Glide)	standard precision
SPME	solid-phase microextraction
VIP	variable importance in projection
vdW	van der Waals
