# Supplementary material for: Integrated Molecular Informatics and Sensory-Omics Study of Core Trace Components and Microbial Communities in Sauce-Aroma High-Temperature Daqu from Chishui River Basin
Source: Foods. 2026 Feb 6;15(3):599. doi: 10.3390/foods15030599 (PMC12897286; doi:10.3390/foods15030599)
Supplement: Supplementary file 1 [file foods-15-00599-s001.zip › File S2. Mechanisms by which 14 core trace components activated the olfactory receptor.pdf]

Mechanisms by which 14 core trace components activated the olfactory receptor (detailed interpretation)

As shown in Table 5, acetic acid (docking score = -6.655) showed the strongest binding tendency. Its electrostatic term, glide ecoul, was -14.562. This pattern indicated that stabilization relied more on hydrogen bonding or polar interactions. For Daqu aroma, strongly polar small molecules often corresponded to a backbone signal of fermentative acidity and freshness. High-intensity odor identity was not required. A background of acidity and fermentation maturity was rapidly established in olfaction. The overall aroma was shifted away from raw green notes. A cleaner and more direct fermentative domain was formed. 4-Methylquinazoline (-4.714) and 1,2,3,4-Tetramethoxybenzene (-3.845) also belonged to the high-affinity group. Their glide evdw values were -15.929 and -18.566, respectively. Hydrophobic matching and shape complementarity were therefore favored. These aromatic nitrogen-containing compounds or polymethoxy aromatics often contributed medicinal nuances, woody sweetness, or a warmer aromatic base. When coexisting with the roasted base of sauce-aroma high-temperature Daqu, texture and aftertaste were more likely to be reinforced. Daqu aroma became thicker and more layered. A thin roasted surface impression was less likely to dominate.

Another pathway with strong influence on sauce-aroma high-temperature Daqu style was contributed by sulfur-containing compounds. Dimethyl trisulfide (-3.950, glide evdw = -13.611) and trans-3-Methyl-2-n-propylthiophane (-3.774, glide evdw = -13.695) both showed relatively stable hydrophobically driven binding. Such molecules typically had very low olfactory thresholds. At trace levels, aroma three-dimensionality and diffusion were markedly altered. When docking suggested easier capture by the receptor, high impact on Daqu aroma became more plausible. Subtle mature notes, onion-garlic-like accents, or meaty nuances were supported. Roasted notes shifted from a dry nutty impression toward a more complex mature profile that better matched sauce-aroma systems. 2-Ethenyl-6-methylpyrazine (-3.414, glide evdw = -13.849) showed a similar binding mode with dominant hydrophobic contributions. Pyrazines were more associated with roasted, nutty, and cocoa-like toasted notes. When binding tendency was favorable, roasted recognizability was more readily amplified by the olfactory system. A key portion that best represented sauce-aroma high-temperature Daqu was therefore strengthened.

Molecules in the moderate binding range were more likely to shape the contour of aroma. Hexanoic acid (-2.121, glide ecoul = -5.397, glide evdw = -13.009) combined polar anchoring with hydrophobic fitting. This feature often corresponded to fatty notes, cheese-like nuances, and fermentative fullness. Strong impact like sulfur compounds was not required. A rounder volumetric impression was formed in olfaction. (E)-2-Pentenal (-1.027, glide evdw = -10.705) and nonanal (-0.508, glide evdw = -14.471) more closely resembled brightness contributors. Unsaturated aldehydes and mid-chain aldehydes often expressed green freshness, waxy fatty nuances, or refreshing diffusion. Docking scores were not extreme. In an appropriate release window, clarity could still be increased. The roasted base was less likely to feel heavy. 2-Hexanone oxime more

likely reflected a range caused by alternative binding conformations or different pocket poses. A shared feature was a markedly negative glide evdw value. Hydrophobic embedding therefore remained important. Sensory effects were less likely to determine specific identity. Subtle freshness, herbal tones, or a softened background were more likely to modify the main odor. Aroma edges became smoother. Abrupt impressions were reduced.

Decanal showed a docking score of 1.952, which indicated unfavorable binding. This result did not imply an absence of odor contribution in Daqu. A more reasonable interpretation was that the selected olfactory receptor model did not favor decanal as a ligand. Perception could have been mediated mainly by other receptor subtypes. Improved binding might also have required a different microenvironment or conformational state. This pattern reinforced a key fact. Sauce-aroma high-temperature Daqu aroma was encoded by multi-receptor and multi-molecule synergy. Preference of a single receptor for a molecular class could bias aroma in one direction. Other molecules could supply layers and details through other receptor pathways.

Overall, the 14 core trace components formed a gradient from strong to weak docking scores. Strongly polar binders such as acetic acid more likely provided a foundation for fermentation maturity and fresh acidic notes. Sulfur compounds and pyrazines more likely provided the most recognizable mature and roasted framework of sauce-aroma high-temperature Daqu. Fatty acids and aldehydes adjusted body and brightness. Polymethoxy aromatics and nitrogen-containing aromatic heterocycles further extended aftertaste and increased aroma texture. Next, the specific interaction modes between these trace components and the olfactory receptor protein will be described.

Hexanoic acid entered the olfactory receptor binding cavity with the carboxyl terminus serving as a polar anchor. A directional hydrogen bond was formed between the carboxyl hydroxyl group and Ser75. Nearby polar residues including Thr74, Thr76, and Ser92 created an auxiliary polar microenvironment. This arrangement stabilized the carboxyl orientation and reduced conformational freedom of the ligand in the pocket (Fig. 1a-c). The hydrocarbon chain extended into a hydrophobic region formed by Leu72, Ile91, Ile103, Leu97, Leu106, Phe101, and Phe102. Tight complementarity was maintained mainly through van der Waals forces and hydrophobic packing. Such tight packing and specific contacts of fatty acid ligands in the pocket were considered an important basis for chain length selectivity and activation efficiency. After the carboxyl group was fixed by polar interactions and the hydrophobic chain was compacted, the receptor was more prone to undergo rearrangement of the transmembrane helices and enter an active state. The downstream signaling was then mediated via Gαolf to activate adenylyl cyclase III, increase cAMP levels, and amplify olfactory signaling.

After trans-3-Methyl-2-n-propylthiophane entered the binding cavity, no obvious strong hydrogen bond anchoring was observed. A binding mode dominated by hydrophobic packing was indicated (Fig. 1d). The carbon framework and thiophane ring were enveloped by a hydrophobic pocket formed by Leu70, Ala71, Leu72, Ile103, Leu106, Phe101, Phe102, and Met100. Stable

residence was achieved mainly through van der Waals contacts and shape complementarity. The sulfur atom exhibited high polarizability. Favorable noncovalent contacts could be formed through S/π interactions with aromatic residues. Binding stability and selectivity were therefore enhanced without reliance on strong polar bonds, which was consistent with general principles of sulfur related noncovalent interactions in protein recognition.

For 2-Hexanone oxime, the oxime group acted as a prominent polar anchor at the binding site. The N-OH end was positioned near Glu94 and was more likely to form a directional hydrogen bond or a salt bridge like polar interaction. The ligand orientation in the pocket was therefore constrained (Fig. 1e). Ser92 and multiple Thr residues were located around the oxime group. A microenvironment was formed that supported hydrogen bonding and polar solvation. The oxime dipole was stabilized and conformational fluctuation was reduced. The hydrocarbon chain penetrated into a hydrophobic cavity formed by Leu72, Ala71, Ile103, Leu106, Phe101, Phe102, and Met100. Energetic contributions were mainly provided by van der Waals forces and hydrophobic contacts. Such shape complementarity was considered to increase residence time and enhance receptor selectivity.

(E)-2-Pentenal lacked a strongly hydrogen donating group. Binding stability was therefore supported mainly by van der Waals interactions and shape complementarity within the hydrophobic pocket (Fig. 1f). The hydrocarbon chain was located in a hydrophobic environment formed by Leu70, Leu72, Ala71, Ala66, Ala67, Ile68, Ile109, Ile113, Leu65, Met64, Phe102, and Val148. Tight packing was established and ligand residence was maintained. The aldehyde carbonyl oxygen showed a clear dipole and was positioned toward a more polar region on one side of the pocket. Weak hydrogen bonding or dipolar interactions could occur with ASH69 and nearby backbone atoms. Aldehyde orientation was thereby constrained and recognition selectivity was improved. When the double bond approached the aromatic ring of Phe102, π alkyl contacts could also occur and further stabilized the conformation.

Dimethyl trisulfide provided almost no strong hydrogen bond donor. Binding stability was determined by hydrophobic complementarity together with polarizable sulfur mediated noncovalent interactions (Fig. 1g). The ligand was located in a hydrophobic cavity formed by Phe101, Phe102, Phe93, Phe156, Leu97, Ala95, Pro159, and Met100. Tight van der Waals contacts were formed and conformational freedom was restricted. When the sulfur chain approached aromatic residues, S/π interactions could occur and enhanced selective recognition and residence stability in the hydrophobic environment. A directional S···O chalcogen bond could also be formed between sulfur atoms and the carboxylate oxygen of Glu94. The polar environment provided by Gln99 and Thr98 further stabilized this orientation and promoted a more consistent ligand pose. Such a binding mode that combined hydrophobic compaction with sulfur related directional interactions was often more capable of inducing contraction of the binding cavity and triggering transmembrane helical rearrangement. Sulfur odor signals were therefore more effectively transmitted to downstream pathways.

Ethyl linoleate binding was dominated by hydrophobic interactions. The hydrocarbon chain formed dense van der Waals contacts with Phe154, Phe155, Phe156, Tyr197, Leu153, Leu158, Leu160, Leu161, Leu203, Leu204, Val205, and Phe212. π alkyl contacts with aromatic residues also contributed and led to hydrophobic compaction within the pocket. The carbonyl oxygen of the ester group served as a polar anchor for receptor recognition. It was located near Arg150, Ser152, Thr200, and Ala201. Weak hydrogen bonds, cation dipole interactions, or directional polar coordination could constrain the orientation and improve residence stability and selectivity. An interaction pattern of polar anchoring plus hydrophobic enclosure was indicated and matched the general feature that olfactory receptor binding cavities were predominantly hydrophobic and relied mainly on hydrophobic recognition (Fig. 1h).

Nonanal exhibited a binding mode dominated by hydrophobic enclosure (Fig. 1i). The carbon chain was mainly embedded in a hydrophobic cavity formed by Leu160, Leu161, Ile162, Pro159, Phe156, Phe102, Phe101, Leu97, and Ala95. Continuous van der Waals contacts were formed and internal motion in the pocket was restricted. The aldehyde carbonyl oxygen was located near Glu94, Thr98, and Gln99. Polar residues in this region could provide dipolar coordination and opportunities for weak hydrogen bonding. Carbonyl orientation was therefore calibrated and recognition selectivity was increased. Overall, such medium chain aliphatic aldehydes were often bound through non directional hydrophobic interactions that enabled plastic binding. The fit of chain length and shape to the receptor pocket was considered a decisive factor. The tendency of the receptor to transition from a resting conformation to an active conformation was thereby influenced.

Compared with Nonanal, Decanal had a longer chain. The pose showed deeper occupation of the hydrophobic channel (Fig. 1j). Dense van der Waals packing was formed mainly with Leu70, Ala71, Leu72, Ile103, Leu106, Ile91, Pro78, Met77, and Leu97. Hydrophobic contacts were also formed with Phe101, Phe102, and Met100. The aldehyde end was located near polar sites including Ser92, Thr98, and Gln99. No explicit hydrogen bond was indicated, yet the carbonyl dipole was more likely stabilized in this microenvironment and orientation drift was reduced. Because olfactory receptor binding cavities were generally hydrophobic, the degree of compaction of long chain aldehydes often determined residence time and activation efficiency. If the chain was too short, contacts were insufficient. If the chain was too long, steric crowding and conformational restriction could occur. Selective responses were therefore more likely driven by chain length matching.

4-Methylquinazoline was centered on an aromatic heterocycle. Binding was driven by the π system together with the hydrophobic cavity (Fig. 1k). Hydrophobic support was provided by Ala71, Ile80, Ile91, Leu13, Ala82, Ala95, Cys96, Met77, and Pro78. π alkyl contacts between the fused ring and Phe102 and Ile103 could occur and stable shape complementarity was formed. Two N atoms on the heterocycle served as electron rich sites recognizable by the receptor. They were positioned near a polar environment formed by Thr74, Ser75, Thr76, Thr98, and Gln99. Weak hydrogen bonding or dipolar interactions were therefore possible. Rotational freedom of the aromatic ring was constrained and conformational consistency was improved. Such nitrogen

containing aromatic heterocycles were usually not locked by a single strong bond. Stability was instead accumulated through multiple weak interactions. Local contraction of the binding cavity and conformational rearrangement of the receptor were therefore more likely induced.

For 5-Methyl-2-phenyl-2-hexenal, a typical aromatic hydrophobic driven binding mode was observed in the pocket. The benzene ring formed π π or π alkyl contacts with Phe101 and Phe102. Hydrophobic enclosure was also provided by Ile103, Leu106, Ile109, Leu72, Ala71, and Leu70. High van der Waals stabilization was thereby achieved for the aromatic and hydrocarbon segments. The aldehyde carbonyl oxygen was positioned at a more polar region near the pocket edge. It was located toward ASH69, Ser152, and Gly151. Orientation calibration could occur through the carbonyl dipole and a weak hydrogen bond network. A more consistent aldehyde direction in the receptor was therefore supported (Fig. 1l). This combination of aromatic anchoring and carbonyl directionality often amplified selective tuning of the ligand to the receptor. Efficient recognition and conformational triggering were especially likely when an aromatic residue array was present in the binding cavity.

For 1,2,3,4-Tetramethoxybenzene, the aromatic plane matched the hydrophobic surface of the pocket. Hydrophobic compaction and π alkyl contacts were formed mainly with Phe101, Phe102, Phe156, Leu158, Leu160, Leu161, Ile162, Pro159, Leu97, and Ala95. Residence stability was thereby determined (Fig. 1m). Methoxy oxygen atoms provided multiple weak polar contact sites. Dipolar interactions or opportunities for indirect hydrogen bonding were more likely near the region of Glu94, Thr98, and Gln99. Aromatic orientation could therefore be fine-tuned without disrupting the hydrophobic core. Multiple methoxy substitution increased the volume of the aromatic system and altered electron density distribution. Dependence on pocket shape complementarity and coordination with aromatic residue arrays was therefore increased. A binding mode dominated by accumulated weak interactions was indicated and could impose a sustained bias on receptor conformational equilibrium.

2-Ethenyl-6-methylpyrazine exhibited binding driven by hydrophobic interactions and heterocycle directionality (Fig. 1n). The hydrophobic portion formed van der Waals contacts with Leu97, Ala95, Cys96, Ile91, Met77, Pro78, Met100, and Phe102. The ethenyl side chain extended into the hydrophobic channel and enhanced shape complementarity. Two N atoms on the pyrazine ring acted as hydrogen bond acceptors. They were positioned near the polar boundary formed by Glu94, Ser92, Thr76, Ser75, Thr74, Thr98, and Gln99. Weak hydrogen bonding or dipolar coordination was therefore possible. Ring plane orientation was constrained and receptor selectivity toward such nitrogen containing roasted note molecules was increased. Such heterocycles often achieved efficient recognition through hydrophobic packing for energetic contributions and ring N atoms for directionality. Local rearrangement of the binding cavity and activation were therefore more readily triggered.

Acetic acid binding was centered on strong polar anchoring. A prominent salt bridge or strong hydrogen bond was formed between the carboxyl group and Arg262. An auxiliary hydrogen bond

was formed between the carbonyl oxygen and Asn194. The carboxylate end was therefore firmly fixed at the polar center of the pocket (Fig. 1o). The hydroxyl end was located toward Leu254. The labeled interaction was more likely associated with a backbone involved hydrogen bond or a short range orientational constraint. A stable two-point orientation of the carboxylic acid was thereby supported. The methyl group formed hydrophobic contacts with Val195, Val179, Leu158, Leu199, Ala201, and Ile202 and provided additional positioning stability. Because strong directional interactions provided higher conformational constraints in receptor recognition, such a locking mode that combined a salt bridge with two hydrogen bonds was considered more favorable for producing a clear activation bias at low concentrations.

Propylene glycol binding was dominated by a multi-point hydrogen bond network. The upper hydroxyl group formed a strong anchoring hydrogen bond with Arg262. The lower hydroxyl group formed a second directional hydrogen bond with His180. A clear orientation of the diol in the pocket was therefore established (Fig. 1p). A polar environment around the hydroxyl groups was shaped by His261, Asn194, Gln181, Asp182, and Ser258. Dipolar coordination and water like solvation effects stabilized hydroxyl electron distribution and reduced conformational wobble. The carbon skeleton formed hydrophobic contacts with Leu254, Val195, Val179, Leu158, Leu199, Ala201, and Ile202. Necessary steric complementarity was thereby provided and compensated for the energetic cost of accommodating a polar ligand in a hydrophobic cavity.

Overall, the docking results indicated that key volatiles in Daqu generally followed a recognition logic that combined polar anchoring with hydrophobic compaction after entering the olfactory receptor binding cavity. Molecules bearing a carboxyl group, hydroxyl group, oxime group, or carbonyl group tended to be directionally constrained at the polar boundary of the pocket. Hydrogen bond donor acceptor roles and dipole stabilization were provided by Ser75, Ser92, Ser152, Thr74, Thr76, Thr98, Thr200, Gln99, and Glu94. Charge or hydrogen bond networks were further reinforced when needed by Arg150, Arg262, Asn194, His180, His261, Asp182, Gln181, and Ser258. Ligand orientation was therefore locked and conformational freedom was reduced. The pocket core remained predominantly hydrophobic. A hydrophobic wall was jointly formed by Leu70, Leu72, Leu97, Leu106, Leu153, Leu158, Leu160, Leu161, Leu199, Leu203, Leu204, Leu254, Ile68, Ile80, Ile91, Ile103, Ile109, Ile113, Ile162, Ile202, Val148, Val179, Val195, Val205, Ala66, Ala67, Ala71, Ala82, Ala95, Ala201, Pro78, Pro159, Met64, Met77, and Met100. Continuous van der Waals contacts enabled compaction in the pocket and determined residence time and chain length matching selectivity. Aromatic or heterocyclic systems also formed π related contacts with Phe93, Phe101, Phe102, Phe154, Phe155, Phe156, Phe212, and Tyr197 and further enhanced specificity through shape complementarity. Sulfur containing molecules exploited sulfur polarizability and formed S/π interactions near Phe93, Phe101, Phe102, and Met100. Directional sulfur related contacts with oxygen containing groups of Glu94 were also possible. Stable binding was therefore supported even for weakly polar ligands. More negative docking scores typically indicated more favorable binding trends, yet interpretation was better suited for relative ranking within the same system. After polar

anchors-imposed directionality and hydrophobic regions achieved compaction, the transmembrane helical bundle was more likely to undergo conformational rearrangement and enter an active state. Coupling to Gαolf then activated adenylyl cyclase III, increased cAMP levels, and amplified olfactory signaling. Receptor level encoding of Daqu aroma differences was thereby supported.

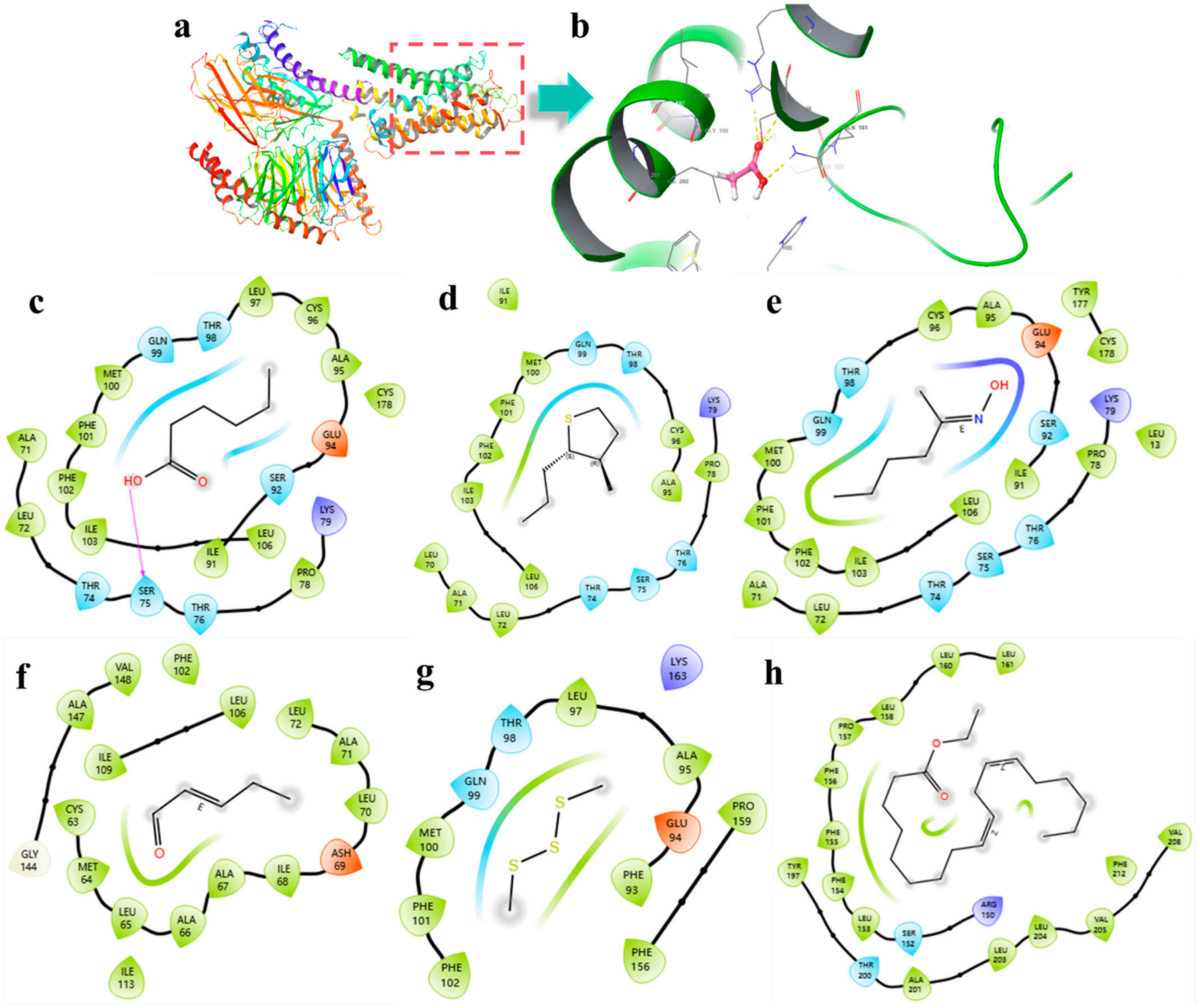
a
b
c
LEU 97
CYS 96
THR 98
GLN 99
ALA 95
MET 100
CYS 178
PHE 101
ALA 71
GLU 94
PHE 102
HO
O
SER 92
LYS 79
LEU 72
ILE 103
LEU 106
ILE 91
PRO 78
THR 74
SER 75
THR 76
d
ILE 91
GLN 99
THR 98
MET 100
LYS 79
PHE 101
S
CYS 96
PHE 102
(S)
(R)
PRO 78
ILE 103
ALA 95
LEU 70
LEU 106
THR 76
ALA 71
THR 74
SER 75
LEU 72
e
TYR 177
CYS 96
ALA 95
GLU 94
CYS 178
THR 98
OH
GLN 99
N
E
SER 92
LYS 79
LEU 13
MET 100
PRO 78
ILE 91
PHE 101
LEU 106
THR 76
PHE 102
ILE 103
SER 75
ALA 71
THR 74
LEU 72
f
VAL 148
PHE 102
ALA 147
LEU 106
LEU 72
ALA 71
ILE 109
E
CYS 63
LEU 70
GLY 144
O
MET 64
ALA 67
ILE 68
ASH 69
LEU 65
ALA 66
ILE 113
g
LYS 163
LEU 97
THR 98
ALA 95
GLN 99
S
PRO 159
MET 100
S
GLU 94
S
PHE 93
PHE 101
PHE 102
PHE 156
h
LEU 160
LEU 161
LEU 158
PRO 157
O
PHE 156
Z
O
PHE 155
VAL 208
TYR 197
PHE 212
PHE 154
Z
ARG 150
LEU 153
SER 152
LEU 204
VAL 205
LEU 203
THR 200
ALA 201

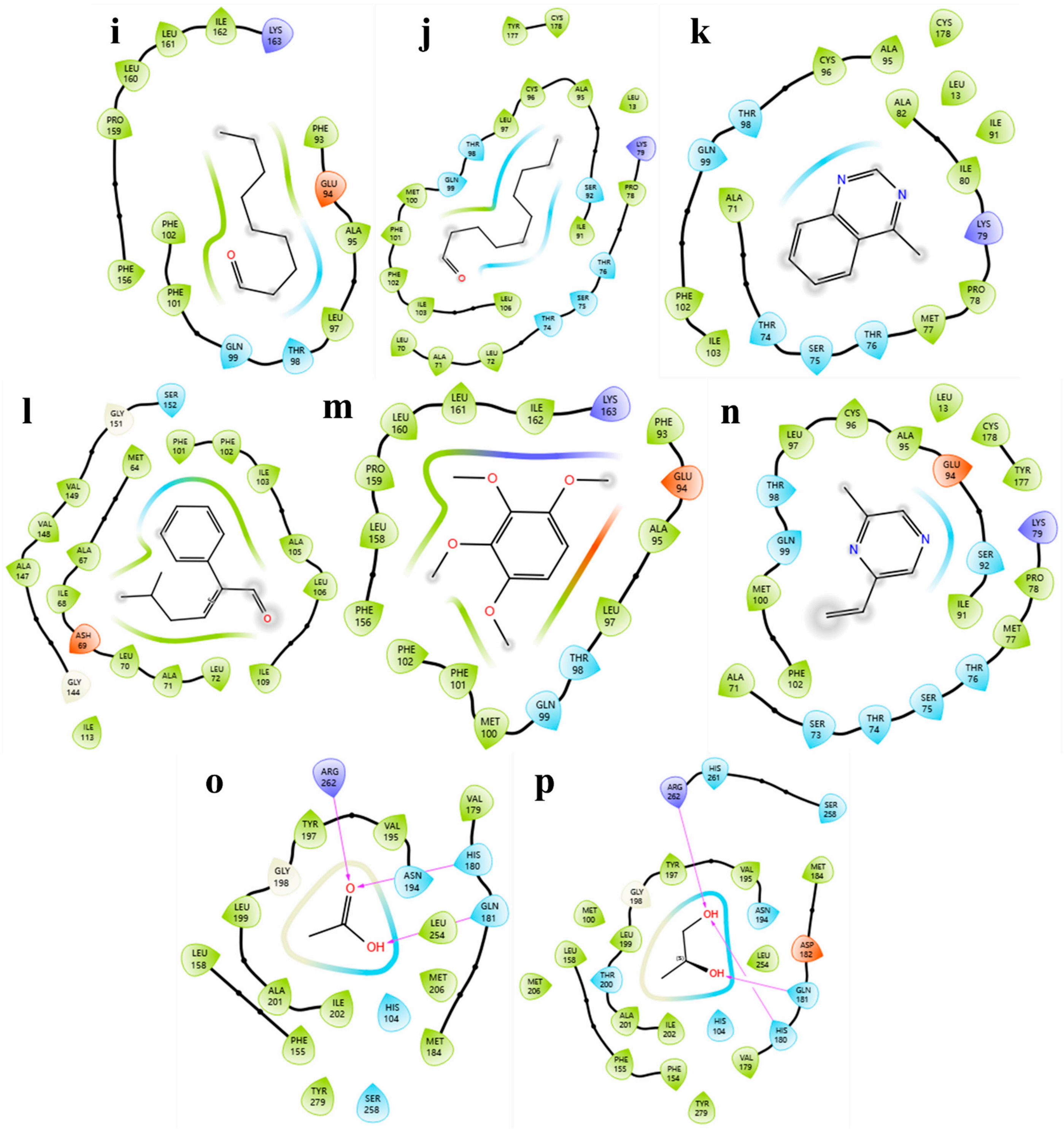

Figure S1. These photos show the olfactory receptor (a) and its microscopic molecular interaction forces (b). The schematic diagrams of interactions between the olfactory receptor and the following compounds are provided in subfigures (c) to (p), respectively: Hexanoic acid, trans-3-Methyl-2-n-propylthiophane, 2-Hexanone oxime, (E)-2-Pentenal, Dimethyl trisulfide, Ethyl linoleate, Nonanal, Decanal, 4-Methylquinazoline, 5-Methyl-2-phenyl-2-hexenal, 1,2,3,4-Tetramethoxybenzene, 2-Ethenyl-6-methylpyrazine, Acetic acid, and Propylene glycol.
