# Supplementary figures and images for: Integrated Molecular Informatics and Sensory-Omics Study of Core Trace Components and Microbial Communities in Sauce-Aroma High-Temperature Daqu from Chishui River Basin
Source: Foods. 2026 Feb 6;15(3):599. doi: 10.3390/foods15030599 (PMC12897286; doi:10.3390/foods15030599)

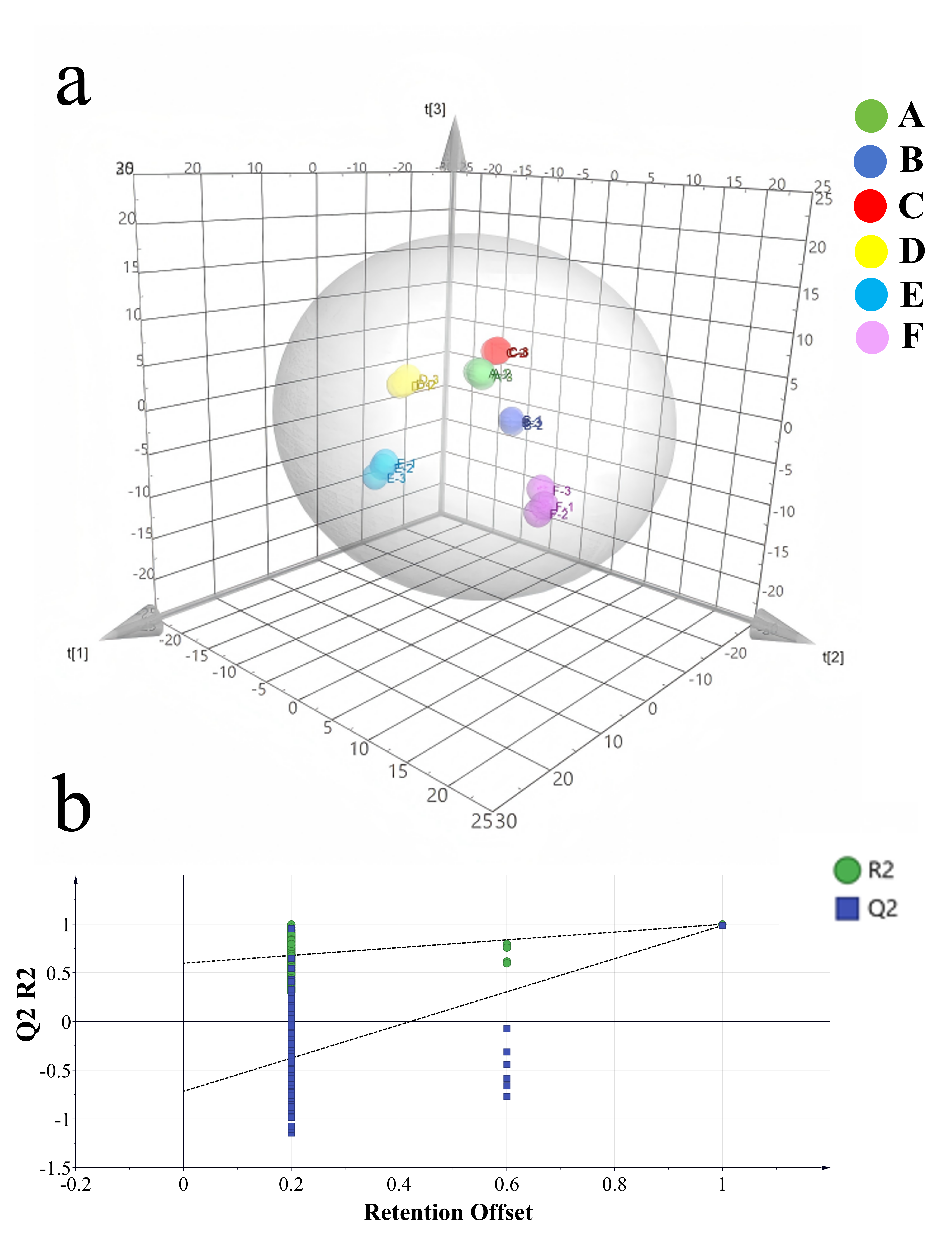

Supplement: Supplementary file 1 [file foods-15-00599-s001.zip › File S1. OPLS-DA of different sauce-aroma high-temperature Daqu samples (a) and model cross-validation results (b).tif]
